# The Effects of Cholesterol Oxidation on Erythrocyte Plasma Membranes: A Monolayer Study

**DOI:** 10.3390/membranes12090828

**Published:** 2022-08-24

**Authors:** Bob-Dan Lechner, Paul Smith, Beth McGill, Skye Marshall, Jemma L. Trick, Andrei P. Chumakov, Charles Peter Winlove, Oleg V. Konovalov, Christian D. Lorenz, Peter G. Petrov

**Affiliations:** 1 Department of of Physics and Astronomy, University of Exeter, Stocker Road, Exeter EX4 4QL, UK; 2 Department of Physics, King’s College London, The Strand, London WC2R 2LS, UK; 3 European Synchrotron Radiation Facility, 71 Avenue des Martyrs, 38000 Grenoble, France

**Keywords:** erythrocyte membrane, lipid monolayers, X-ray diffraction, X-ray reflection, molecular dynamics simulations

## Abstract

Cholesterol plays a key role in the molecular and mesoscopic organisation of lipid membranes and it is expected that changes in its molecular structure (e.g., through environmental factors such as oxidative stress) may affect adversely membrane properties and function. In this study, we present evidence that oxidation of cholesterol has significant effects on the mechanical properties, molecular and mesoscopic organisation and lipid–sterol interactions in condensed monolayers composed of the main species found in the inner leaflet of the erythrocyte membrane. Using a combination of experimental methods (static area compressibility, surface dilatational rheology, fluorescence microscopy, and surface sensitive X-ray techniques) and atomistic molecular dynamics simulations, we show that oxidation of cholesterol to 7-ketocholesterol leads to stiffening of the monolayer (under both static and dynamic conditions), significant changes in the monolayer microdomain organisation, disruption in the van der Waals, electrostatic and hydrophobic interactions between the sterol and the other lipid species, and the lipid membrane hydration. Surface sensitive X-ray techniques reveal that, whilst the molecular packing mode is not significantly affected by cholesterol oxidation in these condensed phases, there are subtle changes in membrane thickness and a significant decrease in the coherence length in monolayers containing 7-ketocholesterol.

## 1. Introduction

Erythrocytes are responsible for delivering oxygen to tissues. Over its lifetime of 120 days, an 8 μm red blood cell must constantly deform in order to flow through capillaries as small as 3 μm in diameter [1]. Any loss in deformability of a red blood cell can have deleterious effects on its ability to flow through blood vessels and oxygenate tissues [2]. As red blood cells are devoid of organelles to regulate their deformability, their mechanical properties are largely determined by the composition and organisation of the plasma membrane and the underlying cytoskeleton [1]. Oxidative stress can lead to the non-enzymatic oxidation of lipids and sterols in the plasma membrane, which is associated with a decrease in deformability of the cell [3]. Oxysterols are present in erythrocytes under physiological conditions, but increased levels of oxysterols lead to apoptosis and are associated with a number of diseases, such as Type 2 diabetes, Huntington’s disease and PKAN [3,4,5,6]. Understanding how the enrichment of oxysterols in the plasma membrane affects the mesoscopic organisation and mechanical properties of red blood cells is essential for the clarification of the molecular basis of these pathologies.

The mammalian plasma membrane is comprised of hundreds of different lipid species, but most contain only a single sterol type, cholesterol [3]. Cholesterol is important for a vast number of cellular processes, including cell signaling [3]. It is vital in mediating the phase of lipid membranes as well as providing mechanical strength to cellular membranes, especially red blood cells [7]. As such, changes to the chemical structure of cholesterol can have significant consequences for the properties of cell membranes.

**Figure 1:**
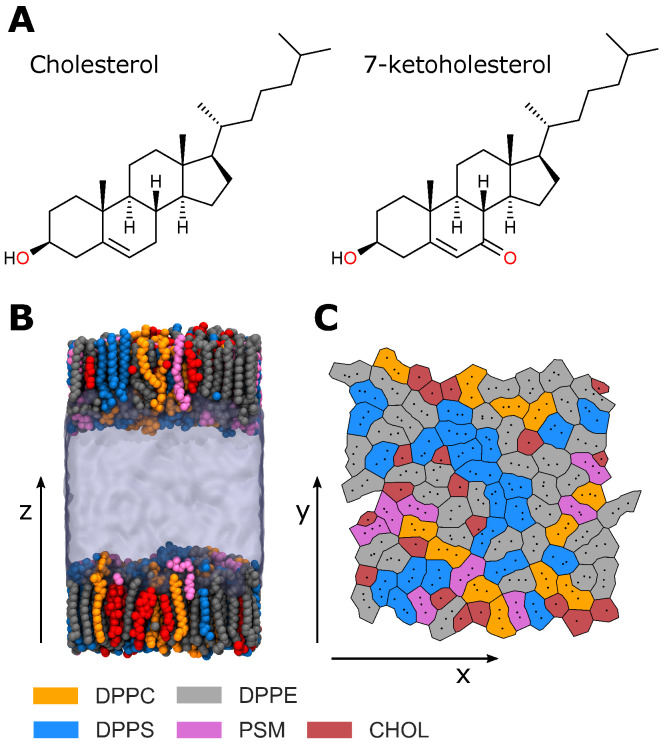
(**A**) Chemical structures of cholesterol and 7-ketocholesterol. (**B**) The RBC Chol monolayer system. (**C**) A Voronoi tessellation illustrating the lateral distribution of lipids in a leaflet.

It has been known for a long time that cholesterol can alter the molecular order, lateral organisation and mechanical properties of lipid membranes and experiments with model lipid systems of varying composition have provided important insights [8,9,10,11]. Much of this discussion has focused on the physical properties of the liquid-ordered phase formed between cholesterol and certain lipids [12] and its relation to lipid rafts in the plasma membrane [13]. When subjected to oxidative stress, cholesterol may react with reactive oxygen species to form ring-oxidised sterols, most commonly 7-ketocholesterol (Figure 1A) and 7β-hydroxycholesterol [3]. Under oxidative stress, unsaturated hydrocarbon lipid tails are more-readily oxidised than cholesterol, but, because the clearance of oxysterols is less efficient than that of oxidised phospholipids, oxysterols are more prevalent than their phospholipid counterparts in the plasma membrane [5]. All healthy cells have small amounts of oxysterols, but many pathologies are associated with a significant increase in oxysterols, especially 7-ketocholesterol [3,5]. The physiological roles of oxysterols include removing excess cholesterol from the body, as well as roles in pro-inflammatory signaling and the metabolism of lipids [3]. However, when present in high concentrations, these oxysterols have been found to have different condensing and ordering effects on phospholipid membranes to cholesterol. Specifically, cellular membranes rich in ring-oxidised oxysterols are more disordered and their lipids are less densely packed [3,14,15]. It may seem counterintuitive that a more disordered plasma membrane results in a less deformable cell, but it has been suggested that disordered domains are the anchor points for actin molecules of the cytoskeleton underneath the lipid bilayer and thus the strengthened interaction of a disordered membrane to the underlying cytoskeleton can lead to increased cell stiffness [16,17,18]. Oxysterols have been found to increase endothelial cell stiffness [19,20] as well as the rigidity of single and multi-component Langmuir lipid monolayers [21,22]. Interestingly, 7-ketocholesterol was the oxysterol that caused the most pronounced effect [20,22]. A recent study of the effect of oxysterols on the stiffness of lipid monolayers mimicking the composition of the erythrocyte membrane and intact red blood cells showed that different oxysterols had opposing effects: whilst 7α-hydroxycholesterol decreased the stiffness, both 7-ketocholesterol and 7β-hydroxycholesterol increased membrane stiffness in these systems, with 7-ketocholesterol having again the strongest impact [23]. We demonstrated previously that 7-ketocholesterol reduces the membrane dipole electrostatic potential in artificial lipid vesicles and T-lymphocytes and influences the interaction of the anti-retroviral agent saquinavir with the multi-drug efflux pump P-gp [24]. It has also been shown that oxysterols can inhibit the formation of liquid–liquid phase separated lipid domains, which will affect the membrane mechanical properties and potentially the ability of the cell to communicate in vivo [14].

Much work has been undertaken to understand the differences between healthy and oxidised red blood cell membranes [2,3,4,5,6,7,14,15,25,26], but there remains a need for an understanding of how oxysterols alter the mechanical and rheological properties of the red cell lipid membrane and its organisation. To reduce the complexity of the system, model lipid monolayers have been used to determine membrane properties [27]. The lateral organisation of the outer leaflet of the erythrocyte plasma membrane has been a subject of a number of studies [13], but the mesoscopic organisation of the inner leaflet remains much less explored. Questions such as differences between inner and outer leaflet physical properties, possible formation of more condensed phases, coupling between microdomains in the outer and inner leaflet etc. remain poorly understood. In particular, it is important to clarify the role of cholesterol in the organisation of the inner membrane leaflet and whether it is involved in formation of microdomains. Here we investigate the effect of cholesterol oxidation on the microdomain structure of synthetic lipid monolayers that are representative of the composition of the inner leaflet of erythrocyte plasma membranes. By means of atomistic molecular dynamics (MD) simulations and experimental surface sensitive methods—including fluorescence microscopy, gracing incidence X-ray diffraction (GIXD), and X-ray reflectivity (XRR)—we provide insight into effect of cholesterol oxidation on the molecular organisation and macroscopic properties of the red cell inner leaflet. We also probe the viscoelastic properties of the monolayers with the aim to clarify the effect of cholesterol and one of its oxidised analogues on the membrane mechanical properties.

A few words about the choice of systems. The vast molecular diversity of lipid species in the membrane makes it virtually impossible to predict its exact macrodomain lateral organisation and physical properties. On the other hand, reducing the system to only two or three components, as often done in model membrane studies, may fail to capture a number of relevant effects resulting from potential synergistic interactions. Our choice for this study is to include the main lipid species found in the mammalian plasma membrane, with a focus on the inner leaflet, a total of five compounds (including cholesterol). As a first step in this approximation, we chose to work with saturated chain lipids for two reasons. Firstly, cholesterol is known to interact with saturated-chain lipids to form raft-like phases, so this system can be seen as probing such interactions and condensed phases. Secondly, including lipids with a wider distribution of chain saturations would significantly complicate the interpretation of results even for a five-component system. Once we clarify the effect of cholestrol oxidation in this system, it could readily be upgraded to include a more realistic distribution of chain lengths and saturations.

## 2. Materials and Methods

We have used a combination of all-atom molecular dynamics simulations and a range of experimental biophysical techniques to investigate the effect of cholesterol oxidation on the inner leaflet of red blood cell model membranes (RBC IL). We use the atomistic simulations to provide an insight into the molecular mechanisms that govern the organisation and properties of the membrane, thus helping the interpretation of experimental observations.

The lipid composition of the studied monolayers approximates that of the RBC IL [28], comprising DPPE (1,2-dipalmitoyl-*sn*-glycero-3-phosphoethanolamine), DPPC (1,2-dipalmitoyl-*sn*-3-phosphocholine), PSM (*N*-palmitoyl-d-erythro-sphingosylphosphorylcholine), the sodium salt of DPPS (1,2-dipalmitoyl-*sn*-glycero-3-phospho-l-serine) and cholesterol (Chol) (Table 1). To determine the effects of cholesterol oxidation, we studied monolayers with no cholesterol content (RBC NChol), monolayers in which all cholesterol was replaced by 7-ketocholesterol (RBC KChol), and monolayers containing native cholesterol (RBC Chol).

### 2.1. Experimental Characterisation of Langmuir Monolayers

All lipids were purchased from Avanti Polar Lipids Inc. (Alabaster, AL, USA) as 1 g/L stock solution in chloroform (total concentration) and used without further treatment.

#### 2.1.1. Static Mechanical Properties

The Langmuir trough technique was used to record surface pressure–area (π-$A_{\mathrm{mol}}$) isotherms of the lipid monolayers at the air-water interface. To create a stable monolayer, an aliquot of the RBC IL lipid mixture solution in chloroform was carefully spread dropwise onto a fresh and clean surface of ultrapure water (Arium mini plus UV, Sartorius, Surrey, UK) or an aqueous Tris buffer solution (100 mM 2-Amino-2-(hydroxymethyl)propan-1,3-diol, Sigma Aldrich Co., Gillingham, UK) at pH = 7, containing 150 mM NaCl and 50 mM CaCl_2_ (Sigma Aldrich Co.) in a Teflon trough (MT-XS, Kibron, Helsinki, Finland, maximal area 110 cm${}^{2}$), using a gas-tight syringe (Hamilton, Hoechst, Germany). The trough was thoroughly cleaned by incubating with a Hellmanex solution (10% in water, Fisher Scientific, Loughborough, UK) and rinsing with copious amounts of water prior to each experiment. To avoid vibrations, the trough was based on an optical table.

After spreading the chloroform solution onto the maximal trough surface, the nascent monolayer was given 20 min to allow the chloroform to evaporate. A Perspex hood was applied throughout to prevent dust falling on the surface and to minimize water evaporation. Regarding the latter, additional water reservoirs not connected to the trough were placed under the hood. The subphase temperature was kept constant at (20.0±0.1) ${}^{\circ }C$ using a circulating bath thermostat (Haake F3, Thermo electron corp., Karlsruhe, Germany). The surface pressure of the film was recorded using a Pt wire probe that had been flamed with a blow torch for cleaning. Two initial compression-expansion isocycles, 0 to 40 mN$m^{-1}$, were recorded at a constant speed of 2 ${Å}^{2}$${\mathrm{molecule}}^{-1}$$\min^{-1}$. Experimental π-$A_{\mathrm{mol}}$ isotherms were recorded at least three times, from which the static compression modulus ε(π) was calculated according to

ε(π)=−A(dπ/dA)

where *A* is the molecular area at a given surface pressure π.

#### 2.1.2. Surface Dilatational Rheology

Surface dilatational rheology was used to quantify the viscoelastic behaviour of the lipid monolayers spread on aqueous subphases, by employing a stress relaxation method [29]. The monolayer was prepared and equilibrated by two expansion-relaxation cycles, as described above. The monolayer was then compressed to a surface pressure slightly below the target pressure, $\pi_{0}$, and pre-equilibrated for about 10 to 20 min with slow dynamic regulation of molecular area if necessary, to keep the pressure constant. Subsequently, a quick ( <1 s) compression with small area perturbation $\Delta A/A_{0}⩽5\%$ (where $A_{0}$ is the initial surface area and ΔA the area change) was performed. The reduction in the area induces a slight surface pressure increase to the target pressure $\pi_{0}$ followed by a pressure relaxation period at constant area over time until a new equilibrium, $\Delta \pi_{\infty }$, is reached. The relaxation process can be approximated as a series of decaying exponential functions and the value of the plateau reached (new equilibrium) at the end of the relaxation, $\Delta \pi_{\infty }$:

$$\Delta \pi \left(t\right)=\Delta \pi_{\infty }+A_{1}e^{-t/\tau_{1}}+A_{2}e^{-t/\tau_{2}}+\cdots$$

where $\tau_{1}$, $\tau_{2}$, ⋯ are the relaxation times of the different relaxation processes in the film and $A_{1}$, $A_{2}$, ⋯ are constants weighing the contribution of the individual relaxation processes. The multiple exponential decay function for the surface pressure relaxation can then be Fourier transformed which results in a complex frequency-dependent dilatational modulus, $E^{*}\left(\nu \right)$:

$$E^{*}\left(\nu \right)=\frac{F\left[\frac{d\Delta \pi \left(t\right)}{dt}\right]}{F\left[\frac{d\ln A}{dt}\right]}=G^{\prime }\left(\nu \right)+iG^{\prime \prime }\left(\nu \right)$$

The dilatational elasticity of the surface is characterised by the the real part of $E^{*}\left(\nu \right)$ which is often referred to as the dilatational storage modulus, $G^{\prime }\left(\nu \right)$. The imaginary part of $E^{*}\left(\nu \right)$ corresponds to the dilatational loss modulus, $G^{\prime \prime }\left(\nu \right)$, which in turn is related to the surface viscosity of the lipid monolayer, $\eta_{D}$, according to:

$$G^{\prime \prime }\left(\nu \right)=2\pi \nu \eta_{D}$$

The tangent of the phase angle φ is given by the ratio of the loss and storage moduli:

$$\tan\varphi =\frac{G^{\prime \prime }\left(\nu \right)}{G^{\prime }\left(\nu \right)}$$

and gives information about the predominant nature of the overall relaxation process. For tanφ<1, the relaxation process is predominantly elastic since $G^{\prime }\left(\nu \right)>G^{\prime \prime }\left(\nu \right)$, whereas tanφ>1 would indicate a predominantly viscous process for which $G^{\prime }\left(\nu \right)<G^{\prime \prime }\left(\nu \right)$. We also employed Cole–Cole plots of loss against storage modulus, which could be conveniently interpreted within the standard linear Maxwell solid model [30].

#### 2.1.3. Epi-Fluorescence Microscopy

Lipid mixture stock solutions were mixed with 0.02 mol% of Rh-DHPE (1,2-dipalmitoyl-*sn*-glycero-3-phosphoethanolamine-N-(lissamine rhodamine B sulfonyl), ammonium salt), a fluorescent lipid. The monolayers were prepared and treated as described above. The subphase temperature was kept at (20.0±0.1) ${}^{\circ }C$ using an external circulating bath thermostat (Haake F6), and the surface pressure was detected using a filter paper pressure sensor. Imaging was performed using an Axio Scope A1 Vario epi-fluorescence microscope (Carl Zeiss MicroImaging, Jena, Germany) coupled to a Langmuir trough (264 cm${}^{2}$ maximal area, Riegler & Kirstein, Berlin, Germany). The setup was mounted onto an electronically controlled (MAC5000, Ludl Electronic Products, Hawthorne, NY, USA) X-Y-stage (Märzhäuser, Wetzlar, Germany), which allowed for the movement of a given area of the film with respect to the imaging system. An area of the monolayer was illuminated by a 100 W Hg arc lamp light source through a 50 X long working distance objective (LD EC Epiplan-NEO-FLUA, Karl Zeiss MicroImaging, Jena, Germany) using a filter and beam splitter (Zeiss filter set 81HE: excitation band-pass BP 553/18 nm, beam splitter FT 570 nm, emission band-pass BP 575 nm–640 nm) that is suitable for the rhodamine label. Images were recorded throughout monolayer compression on an EMCCD camera (ImageEM C9100-13, Hamamatsu, Herrsching, Germany) using the software AxioVision (Carl Zeiss MicroImaging).

#### 2.1.4. Surface-Sensitive X-ray Techniques

X-ray Reflectivity (XRR) and Grazing Incidence X-ray Diffraction (GIXD) were used to resolve the lateral and transverse molecular organisation in the lipid monolayers. We used the synchrotron source at the ID10 beamline Surface Scattering Station of the European Synchrotron Radiation Facility (ESRF) in Grenoble, France (experiment number SC-4816) [31]. Both XRR and GIXD are described in detail elsewhere [32,33,34,35,36]. The monolayers were spread, as explained above, on an in-house-made Langmuir trough with a maximal area of 711 cm^2^ and a single movable barrier, mounted on an active antivibration system. After spreading the film, the trough was sealed with a hood filled with He. The trough temperature was kept constant at (20.0±0.2) ${}^{\circ }C$ using a circulating bath (Julabo MA-4, Seelbach, Germany). Monochromated (Si (111) crystal channel cut monochromator) X-ray light at λ=1.55 Å (8 kV, and synchrotron current of 200 mA) with a high photon flux of $2.7\;\times \;10^{12}$ ph s${}^{-1}$ was used for the experiments. The X-ray beam was focused on the sample using 2D compound refractive lenses and formed a spot with a size of 14×140 μm${}^{2}$. Different areas of the monolayer were exposed to the microbeam in each separate experiment to avoid radiation damage to the sample, especially when performing measurements on less mobile monolayers at high surface pressures.

For the XRR experiments, the sample was irradiated under the glancing angle, α, varied by a double crystal deflector. The reflected radiation under specular reflection was acquired using a linear detector (Mythen 1K, 1280 pixels with a pixel size of 8000×50 μm${}^{2}$). This setup enables the detection of variations in the film structure in the *z*-direction (along the normal to the interface), as the wave vector transfer of the reflected X-ray will be entirely along this direction. The monolayer reflectivity, $R(q_{z})$, was then calculated as the deviation from an ideal flat and smooth surface (Fresnel reflectivity, $R_{F}\left(q_{z}\right)$). From these data, electron density curves of the monolayer along the *z*-direction can be extracted [37,38].

For the GIXD experiments, the film was irradiated at an incidence angle of $\alpha_{i}=0.1233^{\circ }$, slightly below the critical angle of pure water at 8 keV X-ray beam energy ($\alpha_{\mathrm{cr}}=0.154^{\circ }$, i.e., $\alpha_{i}=0.8\alpha_{\mathrm{cr}}$). GIXD spectra were acquired using a double linear detector (Mythen 2K, 2560 pixels with a pixel size of 8000×50 μm${}^{2}$) mounted behind a vertically oriented Sollers collimator with an in-plane angular resolution of 1.4 mrad. Diffracted intensity was detected as a function of the vertical, $q_{z}$, and horizontal, $q_{xy}$, scattering vector components [39]:

$$q_{xy}≃\frac{4\pi }{\lambda }\sin\left(\frac{2\theta_{h}}{2}\right),q_{z}≃\frac{2\pi }{\lambda }\sin\alpha_{f}$$

where $\alpha_{f}$ is the out-of-plane scattering angle in the *z*-direction, and $\theta_{h}$ is the horizontal scattering angle in the xy-plane. The in-plane component provides information about lateral crystalline order in the hydrocarbon lipid chains, whereas the out-of-plane component can be used to estimate acyl chain tilting (angle and direction) [34,35,39,40,41]. Analyses were performed using in-house scripts developed at ESRF ID10.

### 2.2. Molecular Dynamics Simulations

A series of all-atom molecular dynamics (MD) simulations have been used to study the effects of cholesterol oxidation on the structural and interfacial properties of the model RBC plasma membrane inner leaflet described above. Simulations of three systems have been performed: one with no cholesterol (RBC NChol), a second with 20 mol% cholesterol (RBC Chol), and a third with 20 mol% 7-ketocholesterol (RBC KChol). Each system contains two monolayers comprised of 100 lipids each (Figure 1B).

#### 2.2.1. Simulation Protocol

The CHARMM-GUI Bilayer Builder was used to generate symmetric RBC Chol and RBC NChol bilayers with 200 lipids per leaflet [42]. The RBC KChol membrane was built by replacing all cholesterol molecules in the RBC Chol bilayer with 7-ketocholesterol. The parameters for 7-ketocholesterol were obtained from ParamChem [43]. For each system, a steepest descent energy minimisation was performed followed by a series of three equilibration simulations using the NVT ensemble with a timestep of 1.0 fs using a Berendsen thermostat at 293 K [44]. During these equilibration stages, the movements of the phosphorous atoms of the lipid headgroups were constrained using position restraints with a force constant of 1000;1000;400 respectively, along with dihedral angle restraints with a force constant of 1000;400;200 respectively. A series of three equilibration simulations using the NPT ensemble was then performed using a timestep of 1.0, 1.0 and 2.0 fs with a Berendsen thermostat and a Berendsen barostat set to 298 K and 1.0 bar, respectively [44]. During the first two of these equilibrations, the movements of the phosphorous atoms of the lipid headgroups were constrained using position restraints with a force constant of 200 and 40 respectively along with dihedral angle restraints with a force constant of 200 and 100 respectively. Simulated annealing, a cyclical process of heating and cooling, was used to improve the lateral mixing of lipids in the bilayers. Over a 50 ns period, the temperature was raised from 293 K to 400 K, then maintained at this elevated temperature for a further 250 ns. The temperature was then lowered to 293 K over a 50 ns period, and the system then equilibrated at this lower temperature for a further 100 ns. To control temperature and pressure, respectively, these simulations used a Nosé–Hoover thermostat [45,46] and a Parrinello–Rahman barostat [47].

The bilayers were then split at the hydrophobic core to produce two monolayers separated by water (Figure 1B). The monolayers are then simulated using a NVT ensemble, which will keep the monolayers at the equilibrated area found from the bilayer simulations as was done in [48,49,50] for 400 ns using a 2.0 fs timestep with a Nosé–Hoover thermostat with a target temperature of 293 K and a time constant of 1.0 ps. All simulations used a cutoff of 1.2 nm for Lennard–Jones forces and a switching function with an inner cutoff of 1.0 nm to ensure the interactions go to zero continuously. Smooth Particle-Mesh Ewald electrostatics were used with a real-space cutoff of 1.2 nm [51,52]. For the monolayer simulations, the unit cell was periodic only in the *x* and *y* dimensions. As such, to produce a pseudo-2D Ewald summation, force and potential corrections were applied in the *z* dimension [53]. All simulations were run using the CHARMM36 force field and the GROMACS 2020.2 molecular dynamics package [43,54,55]. The final 20 ns were used for analysis of the monolayer trajectories. All analysis was performed with in-house Python scripts using MDAnalysis [56,57], LiPyphilic [58], and Freud [59]. The hydrogen bond analysis tool of MDAnalysis was used for finding hydrogen bonds [60].

#### 2.2.2. Analysis Methods

Various structural properties were calculated to characterise the simulated model RBC monolayers. Specifically, the area per lipid, deuterium order parameter, cholesterol orientation, monolayer thickness and electron density profile were calculated. The average area per lipid was calculated for each lipid species in a leaflet via a Voronoi tessellation (Figure 1C) of reference atoms in the lipid headgroups (see Table S1). Three reference atoms were used for each lipid and one reference atom for each sterol to better capture the difference in size between their headgroups [61]. The deuterium order parameter, $S_{\mathrm{CD}}$, is a measure of the degree of order in the lipid acyl tails, given by:

$$S_{\mathrm{CD}}=-\frac{1}{2}\langle 3\cos^{2}\beta -1\rangle$$

where β is the angle between a carbon-deuterium (or carbon-hydrogen) bond in a lipid tail and the bilayer normal, which is taken to be the *z*-axis. $S_{\mathrm{CD}}$ was calculated as a function of carbon position along the hydrocarbon tail for both the *sn*-1 and *sn*-2 tails of each lipid. The values of $S_{\mathrm{CD}}$ range from −0.5–1.0 whereby larger positive values indicate more ordered acyl tails. Total electron density profiles along the bilayer normal were calculated for each of the systems and presented here as averages over the two monolayers in each system. The orientation of cholesterol with respect to the membrane normal was calculated along the molecular axes from C_3_ to C_17_ (Figure S1). The thickness of the acyl tails and lipid headgroups was calculated as the mean distance from the terminal tail carbon to the carbonyl carbon and from the carbonyl carbon to the phosphorous atom, respectively (see Figure S1). The total thickness was calculated by summing the tail and headgroup thicknesses.

We investigated cholesterol–lipid interactions through producing interaction heatmaps that reveal the most common and important interactions between cholesterol and each lipid species. These were produced by counting the total number of times each atom pair is within 4.0 Å of one another, then normalising these counts by the total number of times a given cholesterol–lipid pair were within 4.0 Å of one another. We took a coarser look at these interactions through a consideration of the types of intermolecular hydrogen bonds formed between lipids in the monolayers. Hydrogen bonds were defined using typical geometric criteria: a hydrogen bond is present if there is less than 3.5 Å between donor and acceptor atoms and a donor-hydrogen-acceptor angle greater than 150${}^{\circ }$.

The properties of the lipid–water interface were described by radial distribution functions (RDF, g(r)) and hydration maps of cholesterol. The RDF is a measure of the probability of finding an atom of type fi at a distance *r* from a reference atom of type α. It is given by:

$$g{\left(r\right)}_{\alpha ,\beta }=\frac{\rho {\left(r\right)}_{\alpha ,\beta }}{\rho_{\beta }}$$

where $\rho_{\beta }$ is the average density of β atoms and $\rho_{\alpha ,\beta }$ is the density of β atoms at a distance *r* from α atoms. We also calculated the total RDF of water atoms around the monolayer headgroup region. This involves creating a histogram of the minimum distances of each water molecule to the monolayer surface, normalised by the density of the bulk water. This provides a measure of the total hydration of the membrane. The hydration maps are visual representations of a cholesterol molecule in which each heavy atom is coloured by the probability of it being hydrated, normalised by the total hydration of the molecule. An atom is considered hydrated if there is a water oxygen atom within 4.0 Å of it.

## 3. Results and Discussion

### 3.1. Effects of Cholesterol Oxidation on Membrane Structure: MD Simulations

The presence of cholesterol in the monolayers decreases both the overall average area per lipid and the area per lipid of each lipid species compared to that observed in the NChol (Table 2). In contrast, 7-ketocholesterol (KChol) actually increases the area per lipid of PSM and DPPS molecules, by 6% and 4% respectively, compared to the RBC NChol monolayers. In the cases of DPPC and DPPE, the condensing effect of KChol is consistently smaller than that of Chol. This is consistent with previous findings that KChol has a smaller ordering effect on DPPC and DPPE lipid membranes compared to Chol [3,5,15].

**Figure 2:**
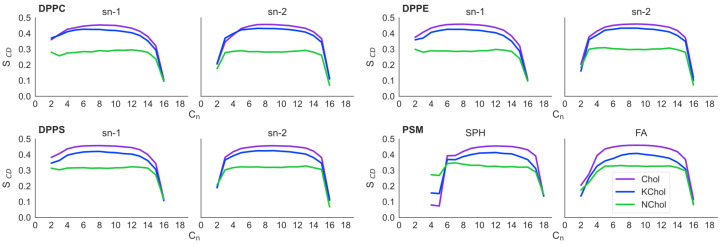
Lipid order parameter ($S_{\mathrm{CD}}$) of the hydrocarbon tails.

The deuterium order parameter ($S_{\mathrm{CD}}$) of each tail in the RBC NChol system is as expected for saturated lipids: more ordered nearer the headgroup region and less ordered at the ends of the acyl tails (Figure 2). This is because the motion of the headgroups is constrained by neighbouring lipids, whereas the tails are more free to move, especially in lipid monolayers [7].

In the RBC Chol monolayers, the value of $S_{\mathrm{CD}}$ of the C_4_ to C_14_ carbon atoms of the saturated tails of each lipid is increased by approximately 0.15 compared to in RBC NChol. In RBC KChol, the value of $S_{\mathrm{CD}}$ of these atoms is also increased compared to in RBC NChol, but by around 0.13, 0.13, 0.11 and 0.09 for DPPC, DPPE, DPPS and PSM respectively. This illustrates the ordering effect that Chol and, to a lesser extent, KChol have on these lipids within the membranes. However, KChol is less adept at inducing order in PSM and DPPS, whilst at the same time it increases the area per molecule of these two lipid species as compared to the NChol membrane.

There is a correlation between area per lipid, $S_{\mathrm{CD}}$ and lipid monolayer thickness. Specifically, as the area decreases, the degrees of ordering and the membrane thickness increase. We see this here with the lipid thicknesses increased in both the RBC Chol and RBC KChol systems compared to the RBC NChol system, but with the effect more pronounced in RBC Chol (Table 3). In both cases, the increased thicknesses are due to the extension of the lipid tails, which is a result of the increased order of the tails induced by Chol and KChol. We find that the increase in the thickness of PSM tails due to Chol is 3.0 times greater than the increase due to KChol. We find that the thickness of the DPPS, DPPC and DPPE lipid molecules in the Chol membrane are 1.5, 1.1 and 1.4 times, respectively, larger than they are in the KChol membrane, which suggests that cholesterol oxidation has the greatest impact on cholesterol–PSM interactions.

The PSM headgroup tends to be more tilted in the RBC KChol system compared to both the RBC NChol and RBC Chol systems (Figure S8), consistent with the reduced headgroup thickness (Table 3). The DPPS headgroup, in contrast, aligns more with the membrane normal in RBC KChol than in the RBC NChol monolayers, and in the RBC Chol monolayers the DPPS headgroup shows greater orientational freedom. There is little difference in the orientation of the DPPC and DPPE headgroups across the three systems.

In RBC Chol, Chol is predominantly aligned with the membrane normal, whereas the KChol in RBC KChol shows a broader angular distribution of orientations (Figure S2). Whilst KChol is also predominantly aligned with the membrane normal, the tendency for it to do so is significantly decreased compared to Chol. As suggested in previous work [15,62], this increased tilt of KChol is likely due to the hydrophilicity of its ketone group, and may explain why it is less capable of condensing and inducing order in lipid membranes.

These molecular level structural differences between RBC Chol, RBC KChol and RBC NChol will affect the lateral mobility of the lipids and thus the mechanics of the monolayers. Below we report on the experimentally-observed mechanical and rheological properties of the interfacial films.

### 3.2. Effects of Cholesterol Oxidation on Monolayer Compression Behaviour: Experiment

#### 3.2.1. Pressure–Area Isotherms

**Figure 3:**
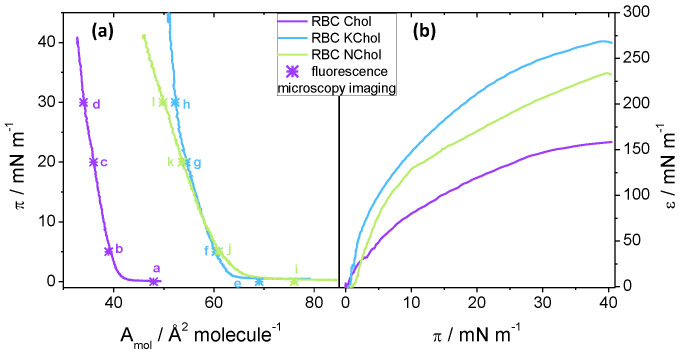
(**a**) Pressure–area isotherms, and (**b**) static compression moduli, ε(π), for the RBC Chol (purple), RBC KChol (blue) and RBC NChol (green) monolayers on a water subphase. Lettered stars mark points in the isotherms where fluorescence micrographs were recorded.

To study the compression behaviour of the films, pressure–area isotherms of the three monolayer mixtures were recorded using the Langmuir trough method. We see that all three mixtures form stable films at the air–water interface upon compression up to 45 mN$m^{-1}$ to 50 mN$m^{-1}$ (see Figure 3).

The isotherm of the RBC Chol monolayer shows a late lift-off from π=0 at 42 ${Å}^{2}$${\mathrm{molecule}}^{-1}$ followed by a steep incline at a rate of about 6 mN$m^{-1}$${Å}^{-2}$molecule until the film collapses (Figure 3a). The cholesterol causes the lipid film to condense and thus considerable repulsive interactions are only present at relatively small molecular areas below ca. 40 ${Å}^{2}$${\mathrm{molecule}}^{-1}$. When no cholesterol is present, there is no condensing effect and the lift-off area is much larger at 68 ${Å}^{2}$${\mathrm{molecule}}^{-1}$ for the RBC NChol system (Figure 3a). Replacing the Chol by KChol—the oxidised variant—leads to a state in between, suggesting that the lipid condensing effect of KChol is smaller than the effect of natural Chol, which is qualitatively similar to the behaviour observed in MD simulations. Somewhat surprisingly, whereas the areas per molecule recorded in experiment for the RBC Chol system are closer to those observed in MD simulations (Table 2), for RBC NChol and RBC KChol systems they are significantly larger. For the RBC KChol system, the lift-off happens at 64 ${Å}^{2}$${\mathrm{molecule}}^{-1}$ upon compression (Figure 3, blue curve in (a)). The RBC Chol film shows a very small area range, of about 10 ${Å}^{2}$${\mathrm{molecule}}^{-1}$, in which non-zero surface pressure is detectable. In contrast, the RBC monolayer without the Chol can be compressed within an area range double that value, whereas the RBC KChol system again shows an intermediate area range of 13 ${Å}^{2}$${\mathrm{molecule}}^{-1}$ between lift-off and collapse. For the RBC NChol mixture, the gradient of the isotherm after the lift-off is 3 mN$m^{-1}$—smaller than the value for the monolayer featuring natural cholesterol. For the RBC KChol mixture, there is a change in the gradient at around 22 mN$m^{-1}$ from 3 mN$m^{-1}$ at π<22 $mNm^{-1}$ (comparable to the value of the RBC NChol system) to 9 mN$m^{-1}$ at π>22$mNm^{-1}$, which is even larger than seen in RBC Chol. This suggests that a phase transition may occur at around 22 mN$m^{-1}$ in the RBC KChol mixture which we verified using fluorescence imaging (see below).

#### 3.2.2. Static Elasticity

The effect of the sterols is also reflected in the quasi-static compressional (dilatational) elasticity of the Langmuir monolayers. For all three monolayers, the static compression moduli ε(π) increase steadily with the surface pressure (Figure 3b). Even for the RBC KChol mixture at around 22 mN$m^{-1}$ the increase is continuous. However, the mechanical properties of the three films upon continuous compression differ depending on the composition, which suggests that the presence of cholesterol and 7-ketocholesterol directly impact the membrane mechanical properties. The RBC Chol monolayer shows the lowest ε(π) value throughout the compression, except at very low pressures (Figure 3b). When cholesterol is removed, the film becomes stiffer, whereas RBC KChol films show the highest static compression modulus of the three monolayers. For instance, at 30 mN$m^{-1}$, $\varepsilon_{\mathrm{RBCChol}}=$ 147 mN$m^{-1}$, while $\varepsilon_{\mathrm{RBCNChol}}=$ 207 mN$m^{-1}$ and $\varepsilon_{\mathrm{RBCKChol}}=$ 250 mN$m^{-1}$. These results suggest that cholesterol oxidation may lead to a considerable degree of stiffening (at 30 mN$m^{-1}$$\varepsilon_{\mathrm{RBCKChol}}=1.7\varepsilon_{\mathrm{RBCChol}}$) in terms of area compressibility under quasi-static conditions.

### 3.3. Effects of Cholesterol Oxidation on Monolayer Dilatational Viscoelasticity: Experiment

**Figure 4:**
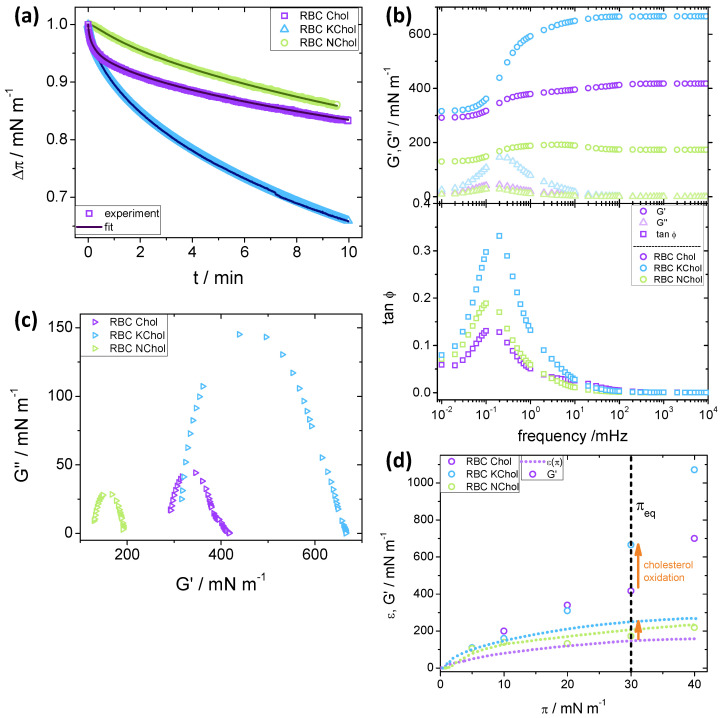
Rheoviscous properties of RBC IL monolayers determined using stress relaxation experiments (dilatational rheology) for the RBC Chol (purple), RBC KChol (blue) and RBC NChol (green) monolayers, at 30 mN$m^{-1}$ and 20 ${}^{\circ }C$. (**a**) Δπ(t) transients (symbols are experimental data, lines are fits), where Δπ is the pressure at a given time, divided by the maximum surface pressure after perturbation $\Delta \pi_{0}\left(t=0\right)$. (**b**) Storage modulus ($G^{\prime }$), loss modulus ($G^{\prime \prime }$) (upper panel) and the tangent of the phase angle ($\tan\phi =G^{\prime \prime }/G^{\prime }$, lower panel) extracted via a Fourier transform of Δπ(t). (**c**) Cole–Cole plots. (**d**) Plot of the plateau of the dynamic storage modulus, $G^{\prime }\left(\pi \right)$ (symbols), along with the static elastic modulus, ε(π), from continuous compression experiments (dotted lines). Data extracted from Figure 3. The arrows show the increase in the moduli due to cholesterol oxidation under quasi-static and dynamic deformation.

We used surface dilatational rheology [63] to probe the dynamic viscoelasticity of the lipid monolayer systems and obtain a more detailed understanding of the effect of cholesterol oxidation. Pre-equilibrated monolayers at a given surface pressure, $\pi_{0}$, were subjected to a small but fast compression to force them out of equilibrium. Subsequent pressure relaxation was recorded at constant area (Figure 4a), and from the Δπ(t) curves the loss, $G^{\prime \prime }$, and storage, $G^{\prime }$, moduli were evaluated as described in the Methods section.

Dilatational rheology experiments were performed at $\pi_{0}=$ 5 mN$m^{-1}$, 10 mN$m^{-1}$, 20 mN$m^{-1}$, 30 mN$m^{-1}$ and 40 mN$m^{-1}$. Below we discuss the general trends for $\pi_{0}=$ 30 mN$m^{-1}$, a representative value for the 2D pressure in lipid bilayers.

For the the RBC Chol system, there is a fast pressure relaxation in the first 30 s–40 s (Figure 4a), which is followed by a slower relaxation process. In contrast, the pressure relaxation for RBC KChol seems to follow a single exponential process. Removing cholesterol from the monolayer altogether results in a much reduced rate of pressure relaxation. As with RBC KChol, the relaxation rate of RBC NChol appears to follow a single exponential process. It should be noted that, overall, RBC NChol shows the smallest surface pressure decrease (15%) upon stress relaxation for the time period investigated (10 min), which is slightly smaller than for the system containing cholesterol (17%). The monolayer with KChol, however, has a pressure relaxation of about 35%, indicating a significant effect of cholesterol oxidation on the dynamic viscoelastic behaviour of the lipid film.

For all three systems studied, $G^{\prime \prime }$ remains smaller than $G^{\prime }$ over the entire frequency range (triangles in Figure 4b, upper panel) and shows a maximum at a frequency of about 0.2 mHz. The height of this maximum is considerably larger for the oxidised system, suggesting that the relaxation of this film has a more viscous nature than the other two. However, the overall relaxation process for all studied monolayers is predominantly elastic, since $G^{\prime \prime }<G^{\prime }$ in the entire frequency region. This is clear from the plot of $\tan\varphi =G^{\prime \prime }/G^{\prime }<1$ (rectangles in Figure 4b, lower panel). For RBC NChol and RBC KChol, $G^{\prime }$ shows an increase coinciding with the maximum of $G^{\prime \prime }$ that leads to a plateau at higher frequencies (circles in Figure 4b, upper panel). For the RBC Chol monolayers, there is a small second rise starting at 100 mHz and leading to a final plateau value, suggesting the presence of two relaxation processes—one fast and one slow. This becomes evident from the Cole–Cole plots [29] in Figure 4c, in which $G^{\prime \prime }$ is plotted against $G^{\prime }$. These plots suggest a single relaxation process for the RBC NChol and RBC KChol system, evident from the single semi-circular trends, whereas a clear deviation is observed for the RBC Chol monolayer. In the context of the generalised Maxwell model of viscoelasticity, the RBC Chol system can be viewed as consisting of two distinctive sets of spring and dashpot elements. Cholesterol has a big effect on the dynamic dilatational elasticity of the monolayer. At higher frequencies, the storage modulus of the RBC NChol system (190 mN$m^{-1}$) is half the value of the RBC Chol system (420 mN$m^{-1}$). Cholesterol oxidation leads to a further increase in $G^{\prime }$ (665 mN$m^{-1}$), indicating a considerable stiffening of the monolayer under dynamic deformation.

Similar stress relaxation experiments for all three monolayers were performed also at target pressures of $\pi_{0}=$ 5 mN$m^{-1}$, 10 mN$m^{-1}$, 20 mN$m^{-1}$ and 40 mN$m^{-1}$. In every case, the loss modulus remains small throughout the experiment, with a faint maximum at low frequencies (around 0.2 mHz). The storage modulus was always higher than $G^{\prime \prime }$, and thus tanϕ<1, again indicating the predominantly elastic nature of the relaxation process for all monolayers at all values of $\pi_{0}$. The Cole–Cole plots indicate a single relaxation processes for RBC NChol and RBC KChol, and a two-step relaxation process for RBC Chol, visible from a shoulder superimposed on main radius of the semicircle. The plateau value of $G^{\prime }$ at high frequencies (above 100 mHz) depends on $\pi_{0}$ and the type of monolayer. This is illustrated in Figure 4d, which shows the $G^{\prime }$ plateau values at different target surface pressures. Data for the static compression modulus, ε(π), are also included for comparison between static and high-frequency dynamic dilatational elasticity.

A comparison of the static and dynamic elastic moduli shows that the three systems exhibit different behaviour. For RBC NChol, the values of $G^{\prime }$ and ε are similar in the entire range of surface pressures investigated. The value of $G^{\prime }$ for RBC Chol is always higher than its static counterpart, and increases steadily up to 700 mN$m^{-1}$ at $\pi_{0}=$ 40 mN$m^{-1}$. RBC KChol exhibits an intermediate behaviour, with $G^{\prime }$ comparable to ε up to $\pi_{0}=$ 10 mN$m^{-1}$, above which the two moduli start to diverge, and at $\pi_{0}=$ 40 mN$m^{-1}$$G^{\prime }$ reaches a very high value of 1070 mNm${}^{-1}$.

The target pressure of $\pi_{0}=$ 30 mN$m^{-1}$ (see the vertical dashed line in Figure 4d) is of particular interest, as this value is representative of the pressure in the cell plasma membrane. Whilst at lower pressures the $G^{\prime }$ values for RBC Chol and RBC KChol are comparable, we see a significant effect of cholesterol oxidation at $\pi_{0}=$ 30 mN$m^{-1}$ (and above) making the KChol containing monolayer much stiffer under dynamic deformation. This may have implications for bilayer membranes with red cell composition. In this pressure range, the storage moduli for these two systems mirror the behaviour of the quasi-static compression moduli, with values for RBC Chol around 60% of those for RBC KChol, indicating that the system containing oxidised cholesterol is much stiffer, both under quasi-static and dynamic deformation, than the one containing natural cholesterol.

### 3.4. Effects of Cholesterol Oxidation on Monolayer Microdomain Structure: Experiment

Static compressibility and dynamic dilatational viscoelasticity measurements reported above serve to inform on the monolayer behaviour as a whole as they produce values averaged over the entire film. However, these monolayers are complex mixtures in which lipid–lipid interactions may drive phase separation on mesoscopic scales. Such microdomain structures are conveniently visualised by epifluorescence microscopy coupled to the Langmuir trough, which allows the recording of images during a continuous compression of the monolayer. For this, a small amount of a rhodamine labelled lipid (Rh-DHPE, dye attached to the head group) was incorporated into the lipid monolayers, as detailed in the Methods section. As the dye moiety is bulky and inflexible, it will be excluded from more densely-packed domains of the lipid film, which in turn will appear dark on the micrographs [64]. Less ordered domains will be bright, since the Rh-DHPE will be accomodated into these regions.

**Figure 5:**
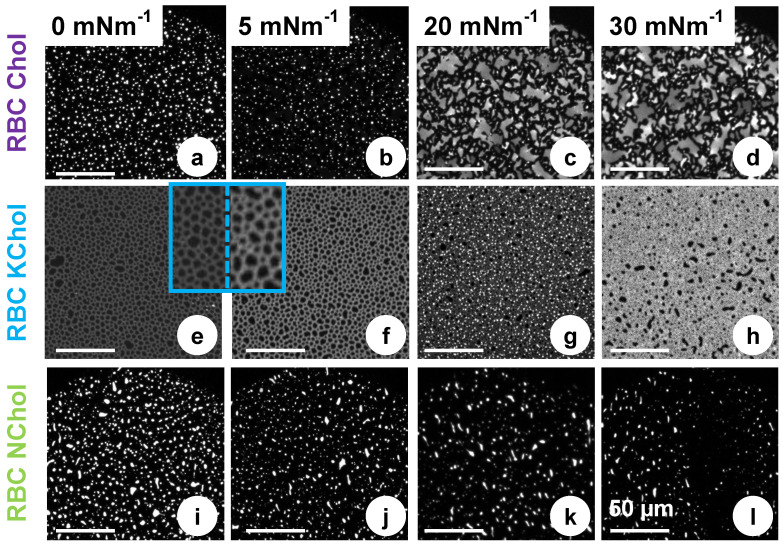
Fluorescence micrographs of the RBC Chol (**a**–**d**), KChol (**e**–**h**) and NChol (**i**–**l**) monolayers on a water subphase at various surface pressures, corresponding to the points marked in Figure 3 (stars). All monolayers contain 0.02 mol% Rh-DHPE. Each scale bar is 50 μm.

Figure 5 shows typical fluorescence micrographs of the three systems investigated. At very low pressures (Figure 5a), the RBC Chol monolayer features small bright domains interspersed in a continuous dark phase. On closer inspection, the bright domains appear round in shape (1 μm–3 μm in diameter), indicating a liquid–liquid phase separation. Upon compression, the abundance of the bright domains is reduced while the overall phase coexistence persists (Figure 5b). Upon compression, large (5 μm–25 μm) irregularly shaped domains of uneven brightness are formed (Figure 5c). To some extent their appearance resembles that of a smectic liquid crystalline phase, and the irregular shapes suggest the domains are solid rather than liquid. This structure persists at higher pressures (Figure 5d) until the film collapses then reforms upon monolayer expansion. From the isotherm, however, we see no clear evidence of a phase transition. However, the pressure at which the new domain structure is detected, ca. 20 mN$m^{-1}$, correlates with the point at which the monolayer storage modulus, $G^{\prime }$, starts deviating from ε, indicating a change in the mechanical compliance of the system.

When cholesterol is removed from the mixture, similar structures are seen at low pressure, i.e., bright domains, 1 μm–8 μm in size, on a dark background (Figure 5i). This phase separation cannot be induced by the condensing effect of cholesterol, and is instead caused by lipid immiscibility, although it is difficult to speculate exactly which lipid species drive this phase separation in a four-component mixture. In contrast to the RBC Chol system, the bright domains are unevenly shaped and feature rough edges, indicating a solid phase. This is supported by the higher static elastic modulus, ε, of RBC NChol compared to RBC Chol (Figure 3b). When the film is compressed, the microdomain organisation remains largely unchanged (Figure 5j,k). This persistence of the same domain structure over the entire surface pressure range seems to correlate with the lack of detectable phase transitions (Figure 3a) and the monolayer viscoelastic behaviour (Figure 4d) which differs from that of the sterol containing systems.

RBC KChol monolayer presents a very different microdomain organisation. At low surface pressures, dark microdomains, 2 μm–8 μm in diameter, embedded in a less ordered continuous phase, are visible in the fluorescence micrographs (Figure 5e). The insets in Figure 5e,f, with a 3-fold magnification, show that the darker microdomains are separated by a network of bright strands containing the fluorescent dye. When compressed to 5 mN$m^{-1}$, the continuous phase appears brighter and thus more enriched in the fluorescent dye (Figure 5f). Upon further compression, the fluorescent lipid appears to concentrate into small bright domains significantly enriched with the dye (Figure 5g). On closer inspection, microdomains of three distinctive grey levels are clearly visible, which makes it likely that at this point a phase separation into three phases with different packing modes and thus different abilities to accommodate the dye-labelled lipid are formed. Although the isotherms do not hint at the presence of a phase transition at this point, 20 mN$m^{-1}$ is the surface pressure that marks the offset of a significant increase of the dilatational storage modulus (Figure 4d). We see a further change in microdomain organisation above 20 mN$m^{-1}$ (Figure 5h), with two visible phases (dark domains embedded in a brighter matrix). At this point the film appears to be very stiff and the fluorescence imaging shows that it is arrested on the aqueous surface with microdomains not able to freely flow past each other as observed at lower surface pressures.

Phase separation in these mixtures is a complex process, driven at the molecular level by the interactions between the lipids. It is clear that this process critically depends on the presence and type of cholesterol, and we find clear parallels between the monolayer microdomain organisation and the monolayer response to mechanical deformation. Although timescales for MD simulations are too short to witness mesoscopic phase-separation, the effects of cholesterol oxidation on lipid–lipid interactions at the atomic level can nevertheless be probed, and these results are presented in the next section.

### 3.5. Effects of Oxidation on Lipid-Lipid Interactions: MD Simulations

**Figure 6:**
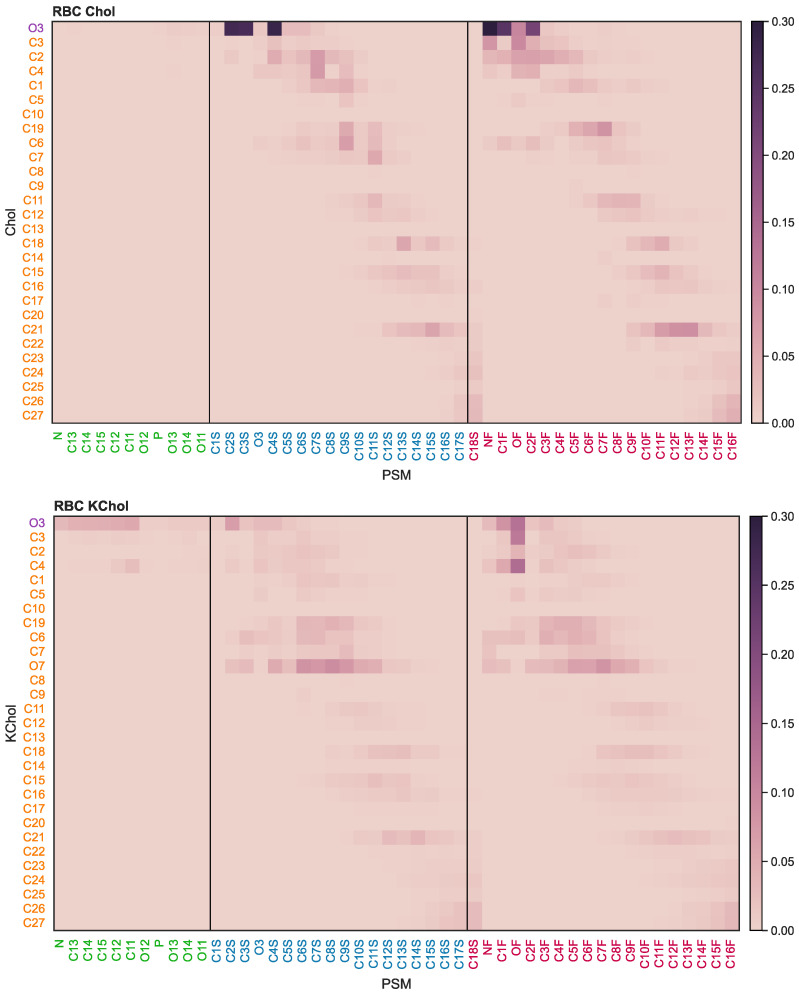
PSM-sterol contact probability. Atom label colours: purple is the sterol headgroup; orange the sterol body; green the lipid headgroup; blue the *sn*-1 tail; and red the *sn*-2 tail.

The oxidation of Chol into KChol leads to a disruption of the van der Waals and electrostatic interactions between the sterol and neighbouring lipids (Figure 6 and Figures S3–S6). The hydrophilicity of the ketone group of KChol leads to a reorientation of the sterol to expose this moiety to the solvent. This disrupts the hydrophobic interactions between the sterol’s carbon rings and the acyl tails of neighbouring lipids as well as the hydrogen bonds between KChol and other lipid species. The disruption of sterol–lipid interactions is most prominent in the case of PSM (Figure 6), which is not surprising given the preferential mixing of cholesterol with sphingolipids over glycerophospholipids [65,66,67]. Further, hydrogen bonds between the amide group of PSM and the hydroxyl group of KChol are 25 times less likely to be found between neighbouring molecules than between PSM and cholesterol (Figure S7). This particular hydrogen bond forms by a PSM molecule straddling the cholesterol molecule, thereby dehydrating its tetracyclic ring structures and preventing the associated free energy cost of solvation [68]. The hydrophilicity of KChol results in the sterol preferentially interacting with surrounding water molecules as opposed to shielding itself underneath a straddling PSM molecule, and therefore disrupts the amide-hydroxyl hydrogen bonds which we have observed occurring between cholesterol and PSM.

### 3.6. Effects of Oxidation on Membrane Hydration: MD Simulations

**Figure 7:**
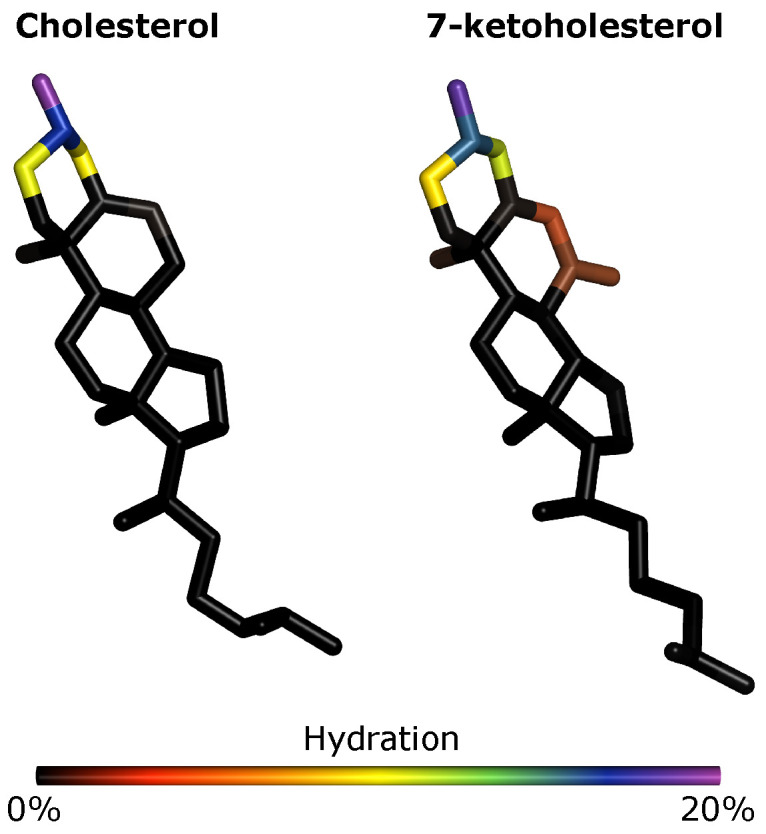
Relative hydration of non-hydrogen atoms of cholesterol and 7-ketocholesterol. An atom rendered purple indicates that 20% of the water within 4 Å of cholesterol is also within 4 Å of that atom.

We see little effect of oxidation both on the total hydration of the membrane (Figure S9) and on which lipids are frequently found in the most hydrated or dehydrated parts of the membrane (Figure S10). However, we do see that the C_7_ and O_7_ atoms of KChol are hydrated whereas Chol C_7_ is dehydrated (Figure 7). We find that C_7_ is 4 times more hydrated in KChol than in Chol and, interestingly, that the hydroxyl oxygen atom of PSM is around 27% more hydrated in RBC KChol than in both RBC NChol and RBC Chol. This means that, not only is the ketone group of KChol hydrated, but more water is also drawn into the headgroup region in the RBC KChol system. This will act, along with the disruption of cholesterol–lipid interactions, to increase the area of the lipids and in turn decrease the order of the acyl tails. There is no significant difference in the hydration of DPPC (Figure S11), DPPE (Figure S12) and DPPS (Figure S13) between the three systems—which is in line with the increased area and decreased order and lipid thickness being most pronounced in the case of PSM (Figure S14).

### 3.7. Effects of Oxidation on the Membrane Interface: MD Simulations

**Figure 8:**
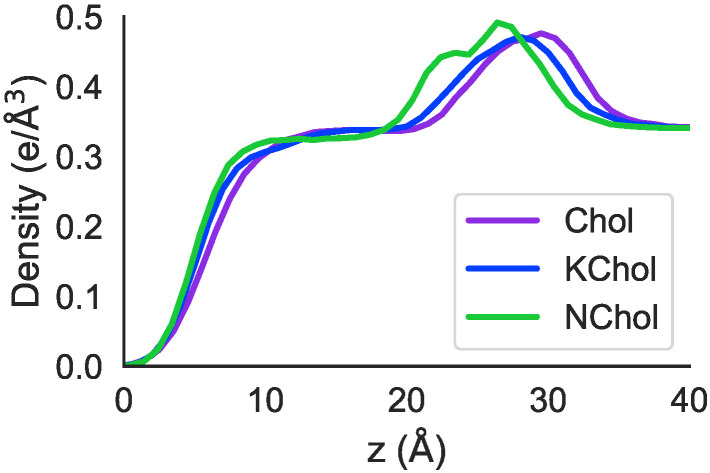
Electron density profiles of the monolayers, derived from the MD simulations.

The peaks in electron density in both RBC Chol and RBC KChol are shifted to larger *z* values compared to in the RBC NChol system (Figure 8), with a greater shift seen in the RBC Chol system. This fits with the earlier observation that—in the MD simulations—RBC Chol is the thickest of the three membranes.

### 3.8. Effects of Oxidation on the Membrane Organisation: Experiment

Electron density profiles were obtained experimentally using surface-sensitive X-ray reflectivity on Langmuir films at the air/buffer interface. For this, we used the soft surfaces and interfaces, high-brilliance undulator beamline (ID10-EH1) at ESRF in Grenoble, France. It should be noted that the X-ray data were obtained using TRIS buffer subphase at pH = 7.4, 150 mM NaCl, 50 mM CaCl_2_ at 20 °C. However, monolayers behave similarly on water and TRIS subphase, as the reflectivity scans and electron densities of the mixtures show no significant dependence on the subphase type (data not shown). Synchrotron X-ray experiments were performed at 5, 15 and 30 mN$m^{-1}$. The reflectivity curves were fitted with a slab model [35,69,70], in which the system is divided into a water slab (of infinite thickness and known refractive index), a head group slab and an acyl chain slab. The parameters to fit were the slab thicknesses, the roughness of the slab interface and the refractive index of the lipid regions.

**Figure 9:**
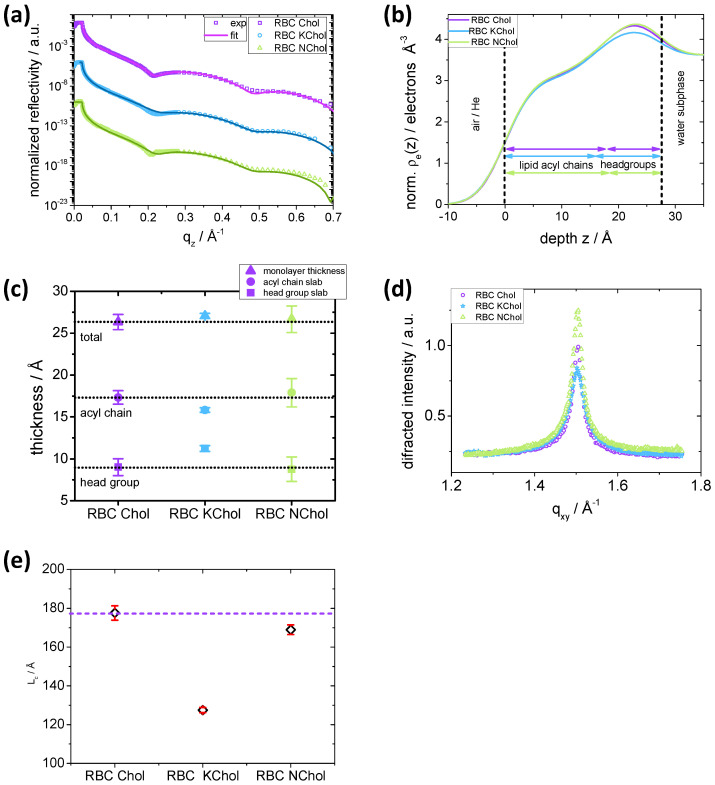
Surface-sensitive X-ray analysis: (**a**) Experimental (symbols) X-ray reflectivity curves of RBC Chol (violet), RBC KChol (blue) and RBC NChol (green) at 30 mN$m^{-1}$ and best fit curves (lines) (the curves are shifted along the *y*-axis for clarity); (**b**) electron density profiles; (**c**) thicknesses of the monolayer and slabs (lipid headgroup, acyl chains, total); (**d**) GIXD curves; (**e**) coherence length (the error bars are given in red). The X-ray experiments were performed using aqueous TRIS buffer at pH = 7.4, 150 mM NaCl, 50 mM CaCl_2_ subphase at 20 ${}^{\circ }C$.

We focus our discussion on X-ray data at 30 mN$m^{-1}$, since this lateral pressure is the most relevant for a comparison with a plasma membrane bilayer. The data at 3 and 15 mN$m^{-1}$ are available in the supplementary information file (Figures S16 and S17). The reflectivity curves, $R_{z}\left(q_{z}\right)$, of all three mixtures have similar features (Figure 9a). The position of the first minimum in the experimental data is slightly different for the three systems, indicating a different thickness of the film comprising the thicknesses of the head group ($d_{H}$) and tail ($d_{T}$) regions. Indeed, the slab model used implies that the position of the first minimum in the normalised reflectivity, $q_{z}^{\left(1\right)}$, is related to $d_{H}$ and $d_{T}$ according to the equation $q_{z}^{\left(1\right)}≃\frac{3\pi }{2}/\left(d_{T}+d_{H}/2\right)$ [39]. For RBC Chol, the first minimum is at 0.2151 Å${}^{-1}$, whereas for RBC NChol $q_{z}^{\left(1\right)}$ is lower, 0.2094 Å${}^{-1}$ and for RBC KChol it is higher, 0.2235 Å${}^{-1}$. The minimum is less pronounced for the oxidised Chol system.

From the normalised reflectivity, we calculated the laterally averaged electron density as a function of the position, *z*, along the normal to the plane of the film. The three monolayers show similar electron density profiles (Figure 9b). The position of the maximum in electron density of RBC Chol is (22.65 ± 0.04) Å, but it is slightly shifted toward the water subphase for RBC NChol and RBC KChol, (23.14 ± 0.04) Å and (22.91 ± 0.04) Å, respectively. This maximum represents the average position of the lipid phosphate group. This group appears to be positioned slightly further away from the tail region for the RBC KChol, indicating a less tilted headgroup moiety. As we saw from the examination of the lipid–lipid interactions, the DPPS headgroup extends further into the water region for RBC KChol compared to RBC Chol. Furthermore, it is noticeable that the overall electron density in the headgroup region is slightly smaller for the system with oxidised Chol.

The analysis of the electron density profile and slab thicknesses reveals that all three monolayers show similar total monolayer thicknesses within the experimental resolution ($d_{\mathrm{tot}}\left(\mathrm{Chol}\right)=\;$(26.3 ± 0.9) Å, $d_{\mathrm{tot}}\left(\mathrm{NChol}\right)=\;$(26.7 ± 1.6) Å, $d_{\mathrm{tot}}\left(\mathrm{KChol}\right)=\;$(27.1 ± 0.3)Å, Figure 9c). However, we see some differences in the thicknesses of the individual slabs. The RBC Chol mixture has a tail slab thickness ($d_{T}\left(\mathrm{Chol}\right)=\;$(17.3 ± 0.8) Å) which is similar to that of the RBC NChol mixture ($d_{T}\left(\mathrm{NChol}\right)=\;$(17.9 ± 1.7) Å). In RBC KChol, however, the tail slab is significantly reduced ($d_{T}\left(\mathrm{KChol}\right)=\;$(15.8 ± 0.3)Å), perhaps due to a larger disorder in the system caused by a reduced condensing effect of KChol on neighbouring lipid tails. In the headgroup region, the opposite trend is visible. RBC KChol ($d_{H}\left(\mathrm{KChol}\right)\;=$ (11.2 ± 0.3)Å) shows a significantly increased headgroup thickness compared to RBC Chol ($d_{H}\left(\mathrm{Chol}\right)=\;$(9.0 ± 1.0)Å) and RBC NChol ($d_{H}\left(\mathrm{NChol}\right)=$ (8.8 ± 1.5)Å). This originates from the fact that KChol is more hydrophilic, situated more toward the headgroup moiety and is able to draw more water from the subphase deeper into the headgroup region. This effect, together with a reduced headgroup tilt, increases the overall headgroup thickness for RBC KChol. The absence of Chol does not seem to affect headgroup thickness.

To investigate the packing of the lipid acyl chains in the monolayers, grazing incidence X-ray diffraction (GIXD) profiles were recorded using synchrotron radiation. All three films show one distinct peak in the GIXD patterns (Figure 9d), indicating that the CH_2_ segments of the stretched acyl chains (π= 30 mN$m^{-1}$) are packed in a hexagonal lattice. Since DPPS and DPPE are the most abundant lipid species in the mixtures, the overall packing mode seems to be dictated by these components, and the acyl chains do not adopt a tilted chain packing typical for DPPC [71].

The peak positions are very similar for all three films ($q_{xy}\left(\mathrm{Chol}\right)=$ (1.50360 ± 9 × 10^−5 ^)Å, $q_{xy}\left(\mathrm{NChol}\right)=$ (1.50360 ± 7 × 10^−5 ^)Å, and $q_{xy}\left(\mathrm{KChol}\right)=$ (1.5031 ± 1 × 10^−4 ^)Å. This indicates the same *d*-spacing of the unit cells (Chol, NChol: 4.179 Å; KChol: 4.180 Å) and thus similar packing modes. From the full width half maximum (fwhm) of the peaks, information on the average domain size can be obtained. Using the Scherrer formula, $L_{c}=0.9\times 2\pi /\left(\mathrm{fwhm}\right)$, the coherence length, $L_{c}$, can be calculated. It is a measure of the average domain size in the direction of the diffraction vector. Relative to RBC Chol ($L_{c}\left(\mathrm{Chol}\right)=$ (178 ± 4) Å), the system without Chol shows a slightly reduced coherence length of $L_{c}\left(\mathrm{NChol}\right)=$ (169 ± 2) Å (Figure 9e). When introducing KChol instead of Chol, the coherence length is significantly reduced to $L_{c}\left(\mathrm{KChol}\right)=$ (128 ± 1) Å. For π= 3 mN$m^{-1}$, no sharp peaks are visible in the GIXD and the halo indicates unordered acyl chains (Figure S17a). At π= 15 mN$m^{-1}$, peaks become detectable for the RBC Chol monolayer giving rise to a weak ordering whereas for both RBC NChol and RBC KChol the peak is barely visible (Figure S17b) indicating reduced acyl chain order in comparison to the monolayer with natural cholesterol.

## 4. Conclusions

The results from this study provide evidence that cholesterol oxidation could have significant implications in terms of membrane organisation (on both molecular and mesoscopic levels), membrane physical properties, and degree of lateral order in lipid monolayers akin to the inner leaflet of the red blood cell. On a molecular level, these effects appear to stem from alterations in the distribution of hydrophilic (headgroup) and hydrophobic (tail) regions of the lipid monolayer containing different sterols as revealed by surface sensitive X-ray techniques. Complementary MD simulations of the same systems suggest that these alterations originate in the significant differences in which cholesterol and its oxidised variant, 7-ketocholesterol, interact with neighbouring lipid species. 7-ketocholesterol, due to its increased hydrophilicity, is able to draw water deeper in the headgroup region of the monolayer thus increasing its size. Oxidation of cholesterol also results in a decrease of the coherence length for these monolayers.

As a first step in understanding of these effects, we chose to work with mixtures of saturated-chain lipid species in order to emulate condensed lipid phases. Whilst our model lipid systems cannot capture the full molecular diversity of the mammalian plasma membrane, they should be representative of the condensed phases in the inner leaflet of the erythrocyte membrane, which are thought to form between cholesterol and saturated-chain lipids. Modifications of their properties as a result of oxidative stress may therefore have effects on such phases if they indeed exist in the inner leaflet, and lead to altered biological function. The obvious example is the membrane mechanical properties, which are of crucial importance especially for the erythrocyte. Increased membrane stiffness in terms of area compressibility (both static and dynamic) reported here complement existing studies that have shown that oxidative stress leads to increase of the membrane bending and shear stiffness [72] and lipid packing [73].

## Figures and Tables

**Table 1:** Monolayer composition (mol %) used to model the inner leaflet of the RBC plasma membrane in the RBC Chol system [28]. In the RBC KChol system, cholesterol (Chol) is replaced by 7-ketocholesterol (KChol). There is no cholesterol in the RBC NChol system.

Lipid	RBC Chol	RBC KChol	RBC NChol
DPPC	12	12	17
DPPE	38	38	43
DPPS	22	22	27
PSM	8	8	13
Chol	20	0	0
KChol	0	20	0

**Table 2:** The mean area per lipid (${Å}^{2}$) of each lipid species. The standard error of the mean—calculated using block averaging over 10ns blocks—is 0.1 or less for each lipid.

	Area Per Lipid/Å${}^{2}$		
**Lipid**	**NChol**	**Chol**	**KChol**
DPPC	48.5	44.7	45.3
DPPE	46.9	43.9	44.8
DPPS	45.0	43.9	46.6
PSM	43.2	41.9	45.9
Chol/KChol	-	21.8	23.8
Average (All)	-	39.4	41.2
Average (Lipid only)	46.1	43.8	45.5

**Table 3:** The mean tail, headgroup, and total lipid thickness (Å). The standard error of the mean—calculated using block averaging over 10 ns blocks—is 0.04 or less for each measurement.

	Lipid Thickness/Å								
	**Tail**			**Headgroup**			**Total**		
**Species**	**NChol**	**Chol**	**KChol**	**NChol**	**Chol**	**KChol**	**NChol**	**Chol**	**KChol**
DPPC	16.1	18.1	17.9	7.6	7.4	7.4	23.6	25.5	25.3
DPPE	16.3	18.2	17.7	6.2	6.1	6.2	22.5	24.3	23.9
DPPS	16.8	18.3	17.8	8.1	7.8	7.9	24.9	26.1	25.7
PSM	17.9	19.4	18.4	6.6	6.6	6.2	24.5	25.9	24.5
All	16.6	18.3	17.8	7.0	6.8	6.8	23.6	25.1	24.7

## Data Availability

All data are available from the corresponding authors.

## Supplementary Materials

The following are available at https://www.mdpi.com/article/10.3390/membranes12090828/s1.
