# Supplementary material for: The Effects of Cholesterol Oxidation on Erythrocyte Plasma Membranes: A Monolayer Study
Source: Membranes (Basel). 2022 Aug 24;12(9):828. doi: 10.3390/membranes12090828 (PMC9506283; doi:10.3390/membranes12090828)
Supplement: Supplementary file 1 [file membranes-12-00828-s001.zip › membranes-1860265-supplementary.pdf]

Supplementary Materials: The Effects of Cholesterol Oxidation on Erythrocyte Plasma Membranes: A Monolayer Study

Bob-Dan Lechner [1,‡], Paul Smith [2,‡], Beth McGill [1], Skye Marshall [1], Jemma L. Trick [2], Andrei P. Chumakov [3], C. Peter Winlove [1], Oleg V. Konovalov [3], Christian D. Lorenz [2,*] and Peter G. Petrov [1,*]

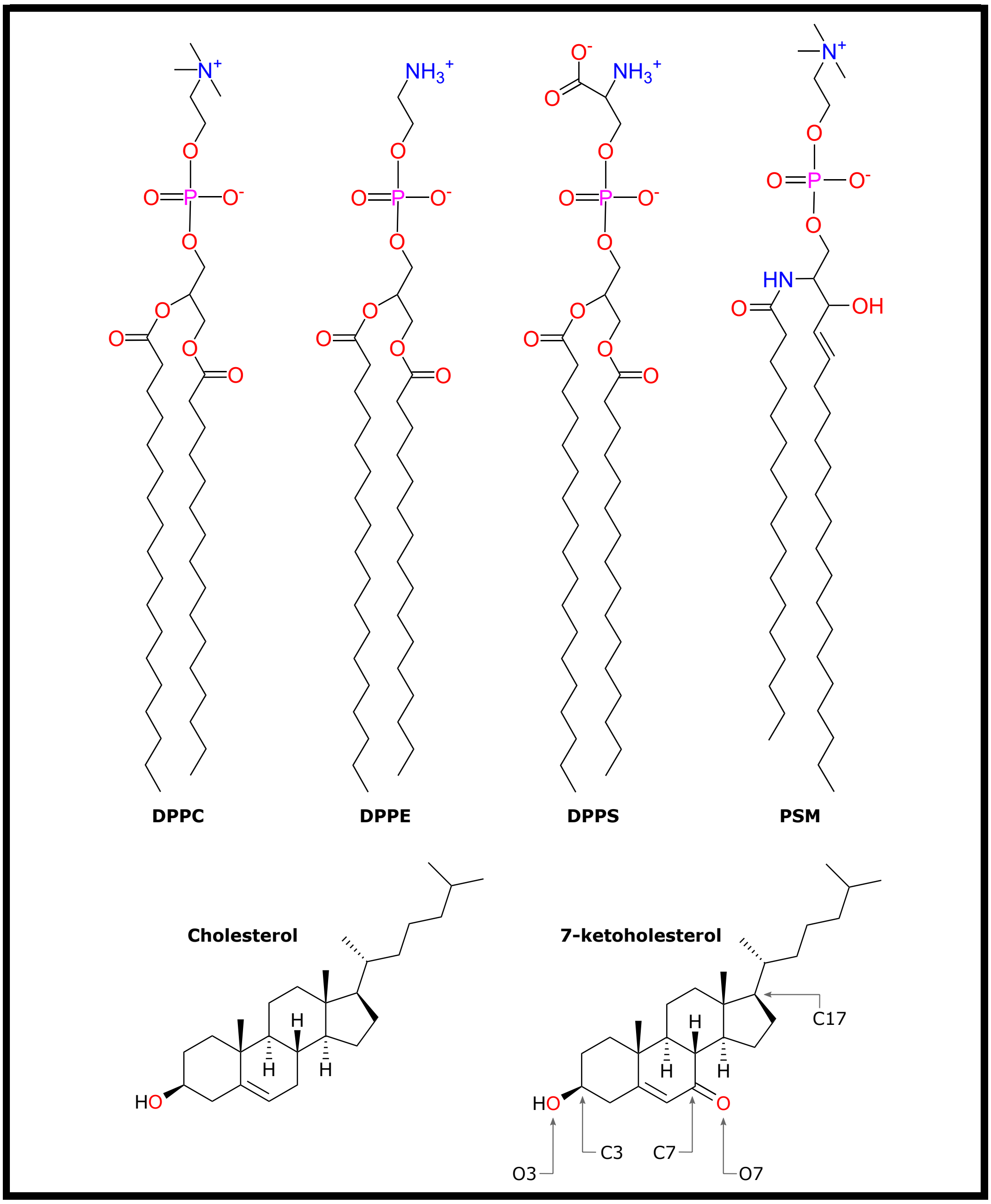

Figure S1. The chemical structures of the lipids and sterols that comprise the model RBC membranes. Chol and KChol atoms used in the analysis of the MD simulations are labelled.

1

Lipid	Reference Atoms
DPPC	C2 C21 C31
DPPE	C2 C21 C31
DPPS	C2 C21 C31
PSM	C2S C1F C4S
Chol/KChol	O3

Table S1. CHARMM atom names of the lipid headgroup atoms used in the Voronoi tessellations of the lipid monolayers.

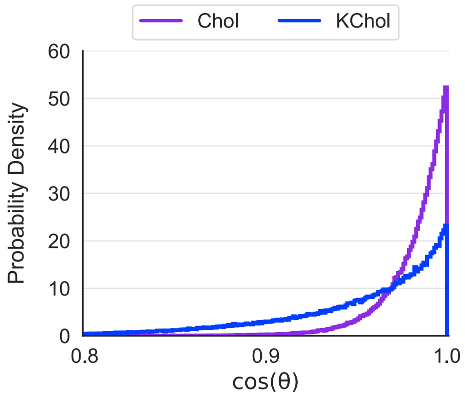

Figure S2. Sterol orientation. θ is the angle that cholesterol makes with the z-plane. The molecular axis of cholesterol is defined as the vector from C_3 to C_{17} (see Fig. S1).

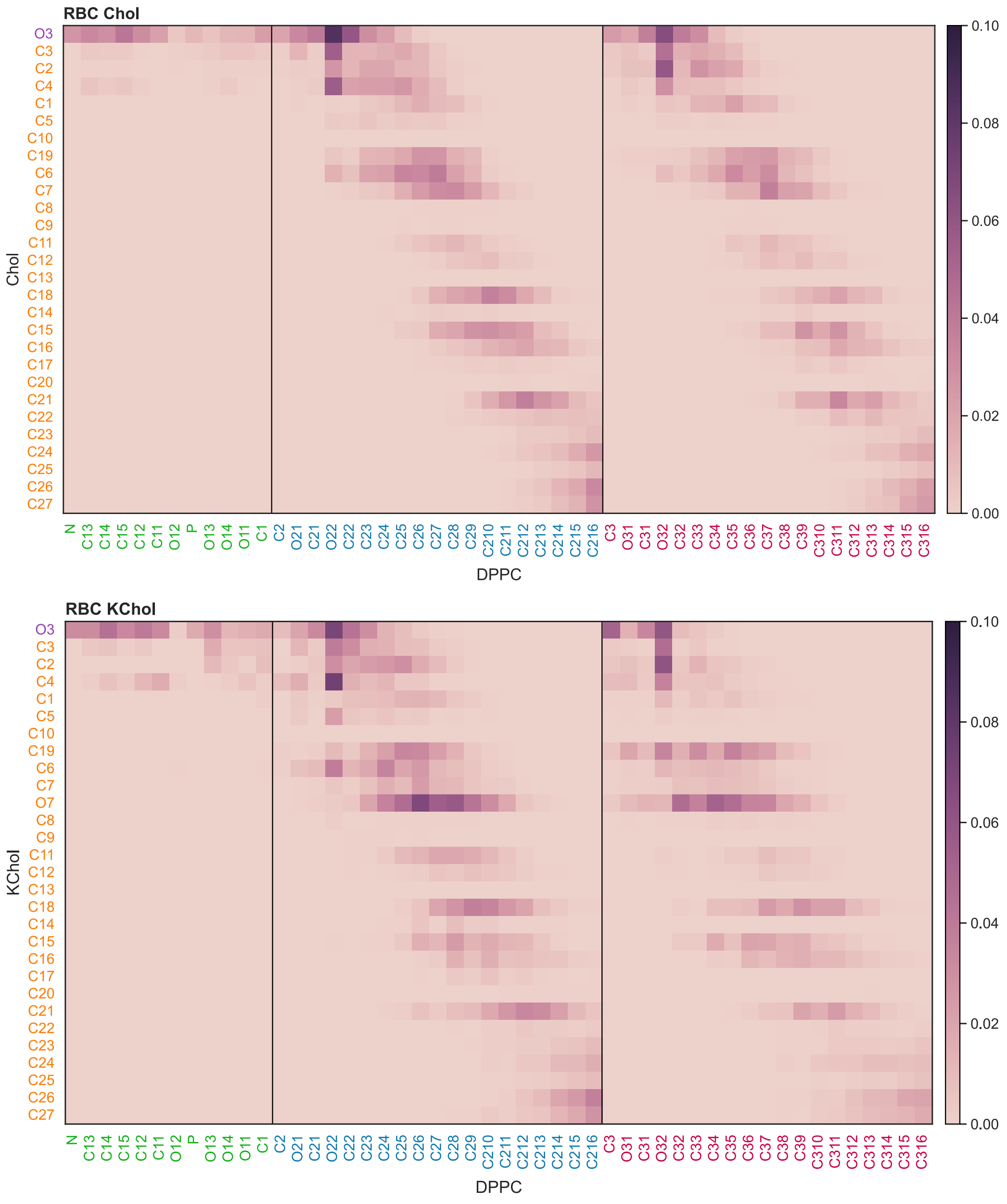

Figure S3. DPPC-sterol contact maps.

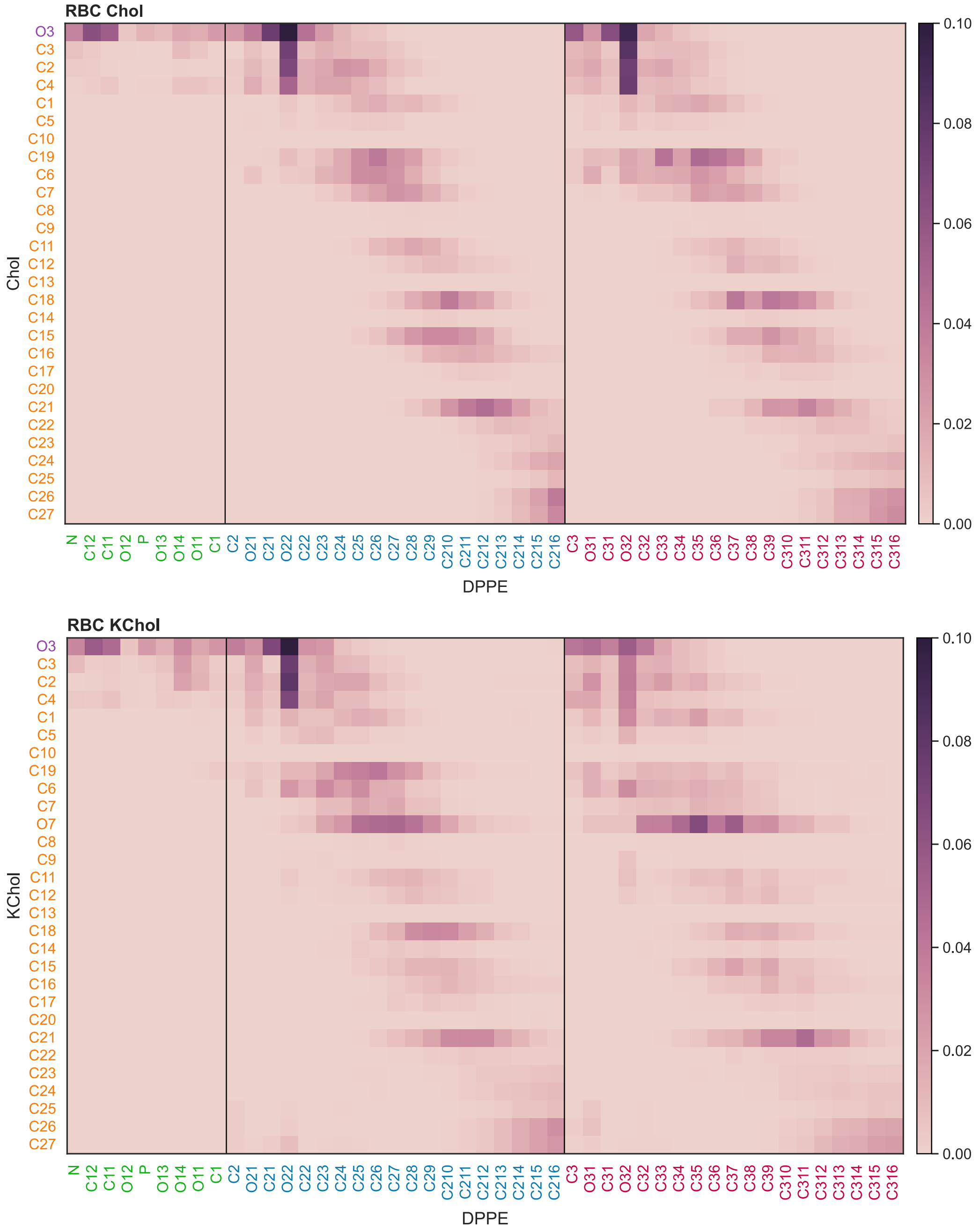

Figure S4. DPPE-sterol contact maps.

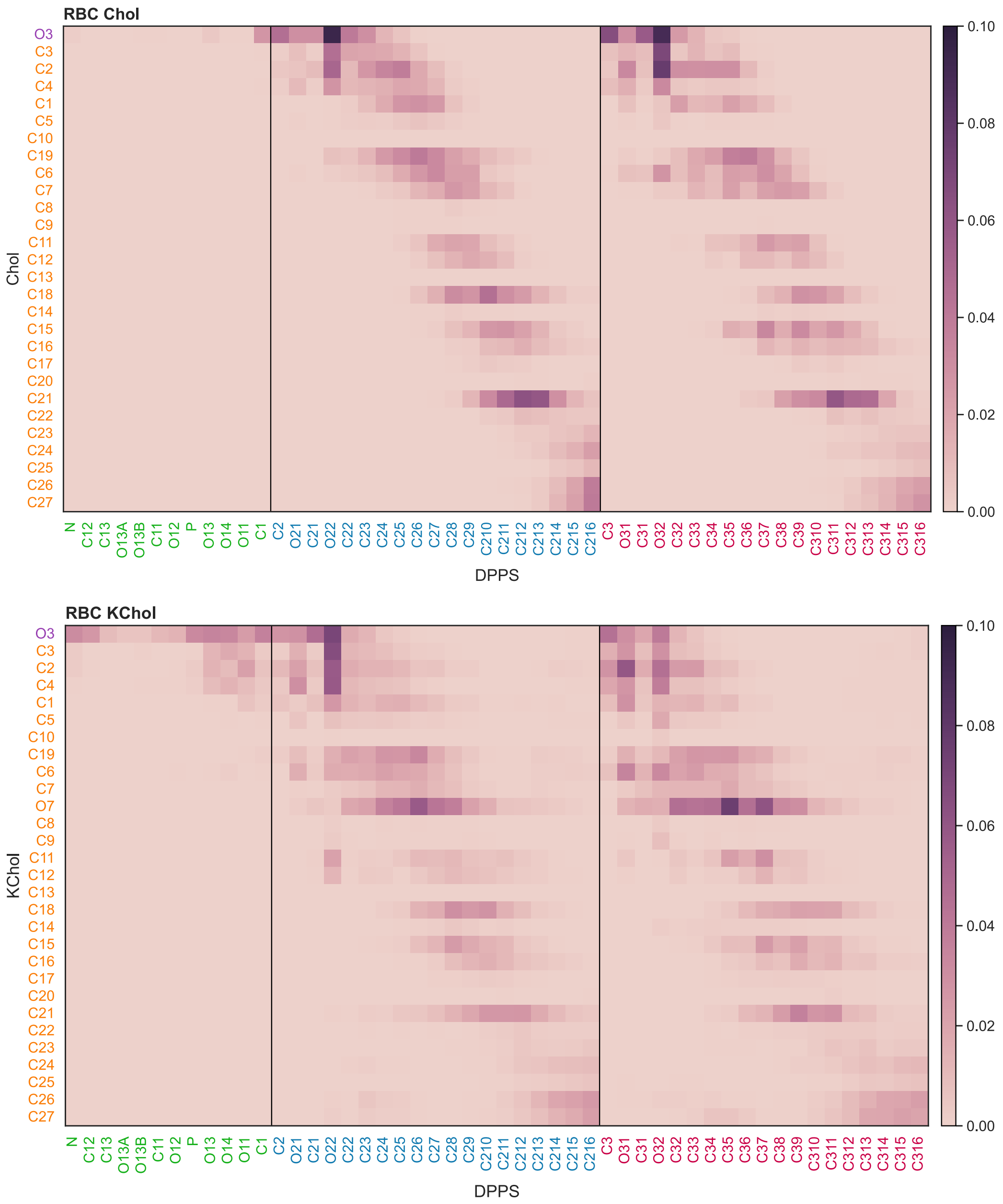

Figure S5. DPPS-sterol contact maps.

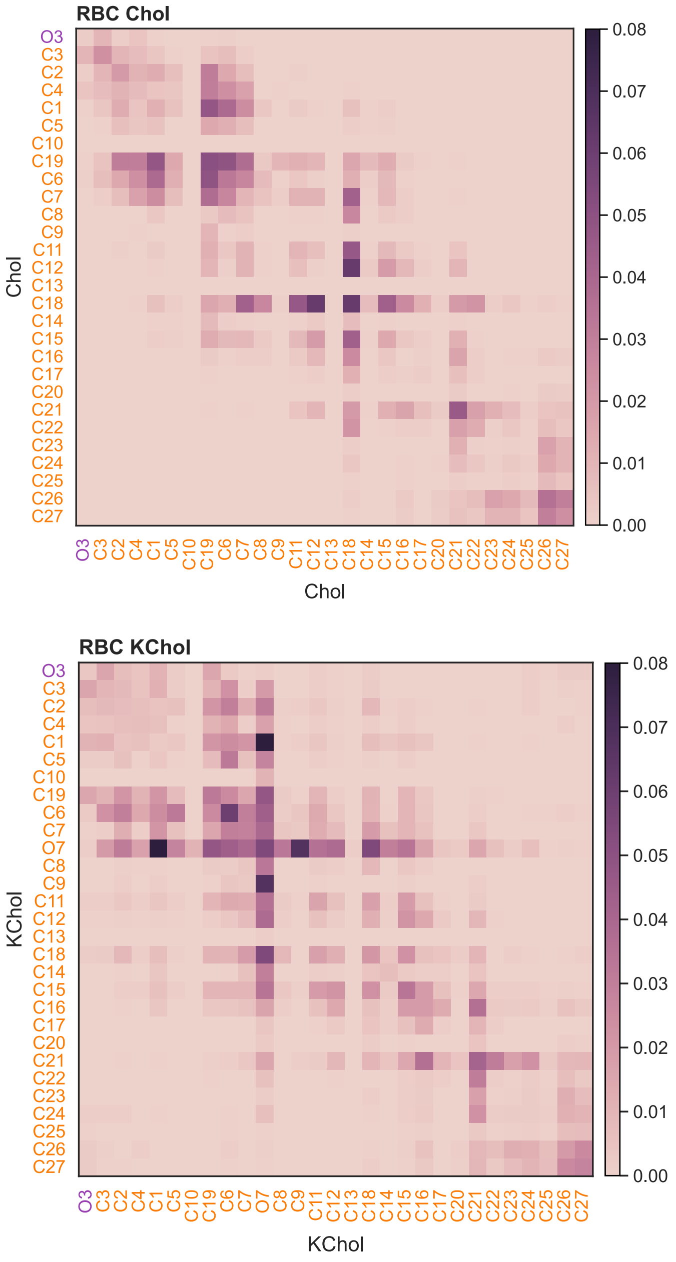

Figure S6. Sterol-sterol contact maps.

Label	Moiety / Element (Lipid)
N	Choline / N (DPPC, PSM)<br>Ethanolamine / N (DPPE, DPPS)
O13, O14	Phosphate / O (DPPC, DPPE, DPPS, PSM)
O13A, O14A	Carboxylate / O (DPPS)
O3	Hydroxyl / O (Chol, KChol, PSM)
O7	Hydroxyl / O (KChol)
NF	Amide / N (PSM)
OF	Amide / O (PSM)
O22, O32	Ester / O (DPPC, DPPE, DPPS, PSM)

Table S2. Atom labels and corresponding lipid moieties / atom elements used in the hydrogen bond analyses.

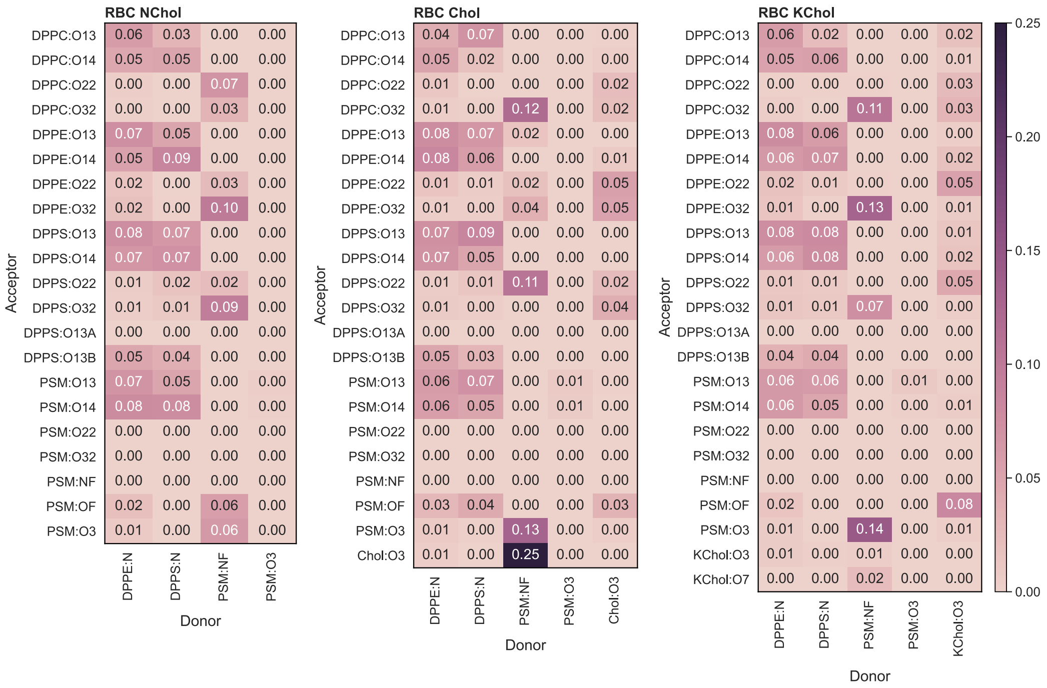

Figure S7. Mean number of each type of hydrogen bonds between neighbouring molecules (acceptor and donor atom labels CHARMM atom names, detailed in Table S2).

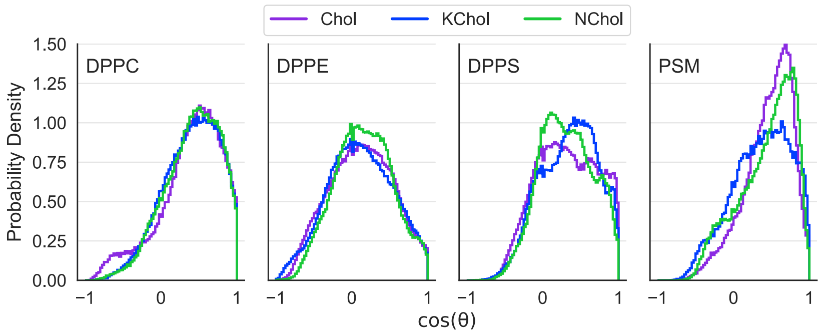

Figure S8. Orientation of phospholipid headgroups, defined as the angle between the headgroup P-N vector and the normal to the monolayer surface.

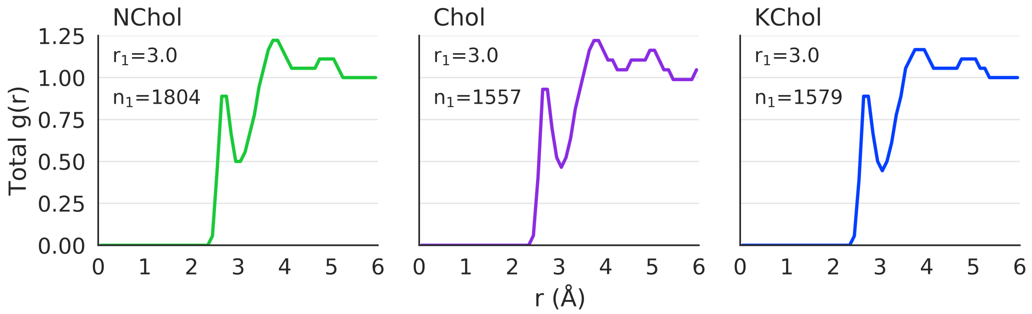

Figure S9. Total radial distribution function of water molecules around the lipid monolayers.

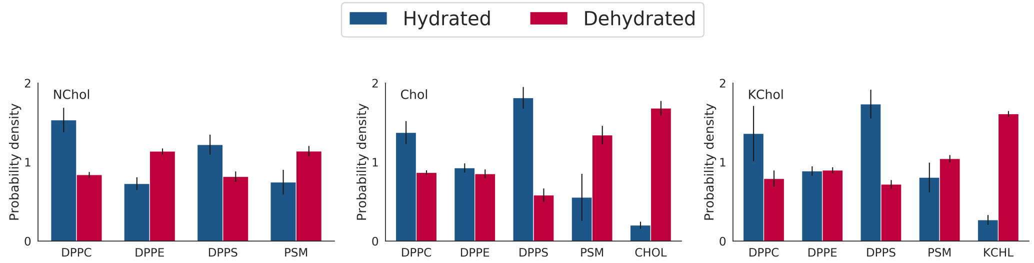

Figure S10. Probability density of finding each lipid species in the 20% most hydrated (blue) or dehydrated (red) regions of the membrane, for the RBC NChol, RBC Chol and RBC KChol systems. The probability is normalised by the fractional composition of the membranes.

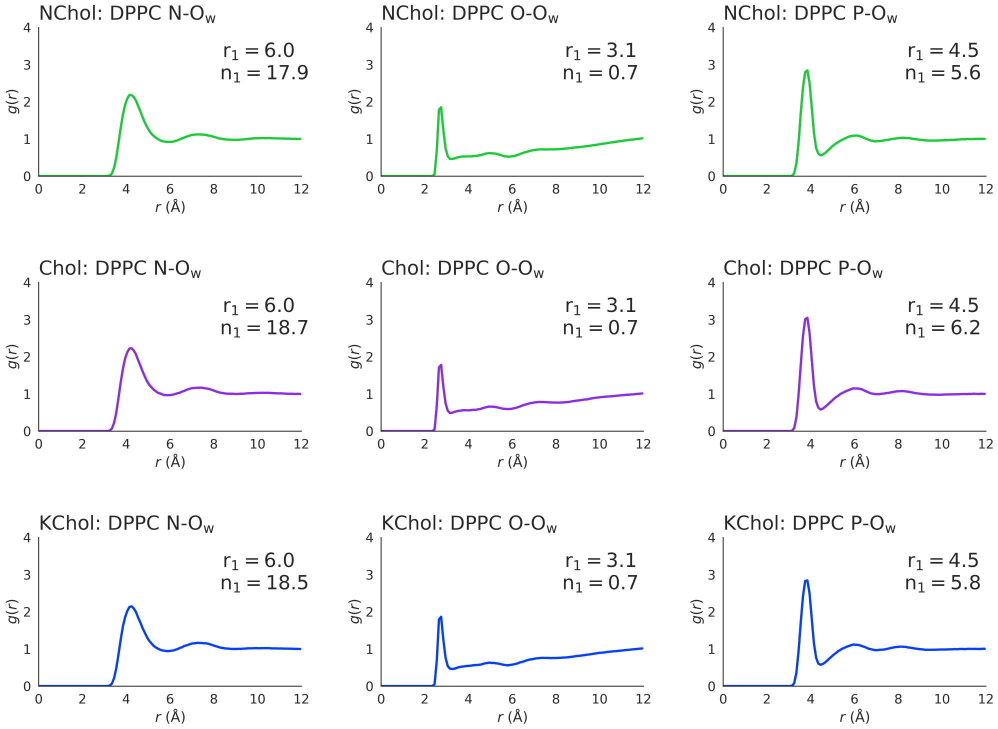

Figure S11. RDF of O_W atoms around the polar atoms (N, P, O) of DPPC.

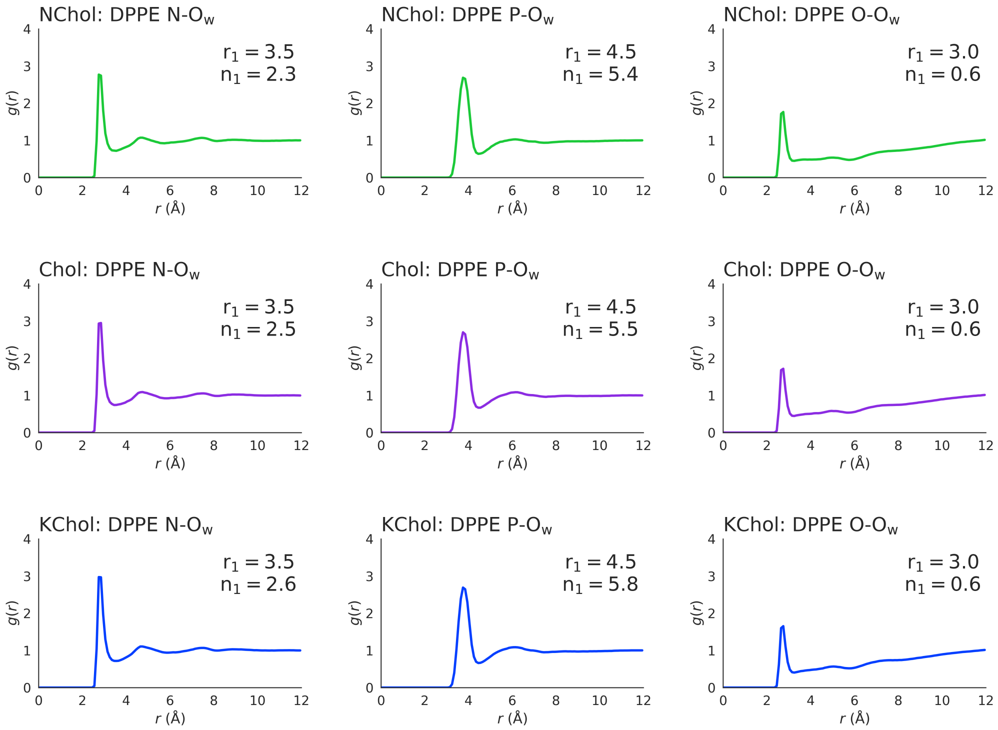

Figure S12. RDF of O_W atoms around the polar atoms (N, P, O) of DPPE.

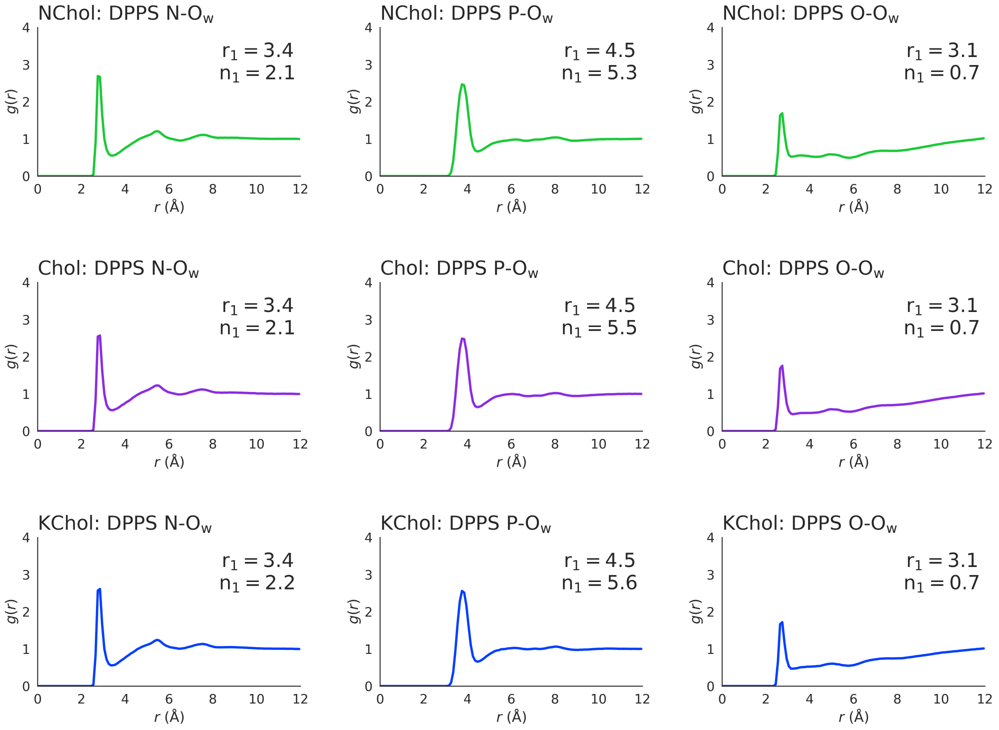

Figure S13. RDF of O_W atoms around the polar atoms (N, P, O) of DPPS.

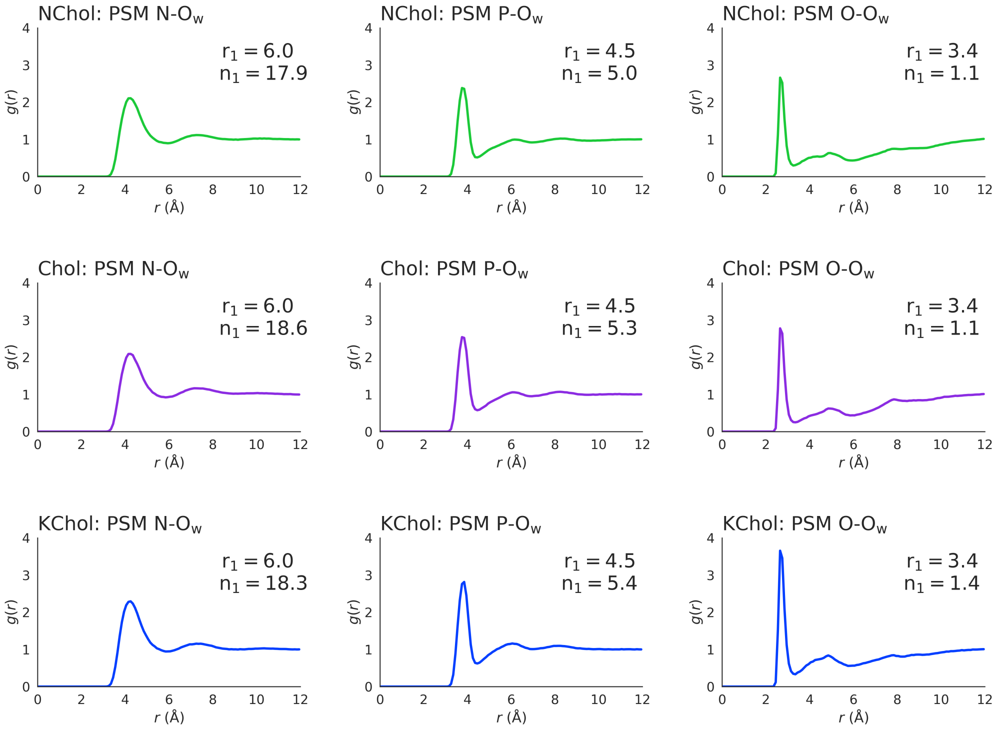

Figure S14. RDF of O_W atoms around the polar atoms (N, P, O) of PSM.

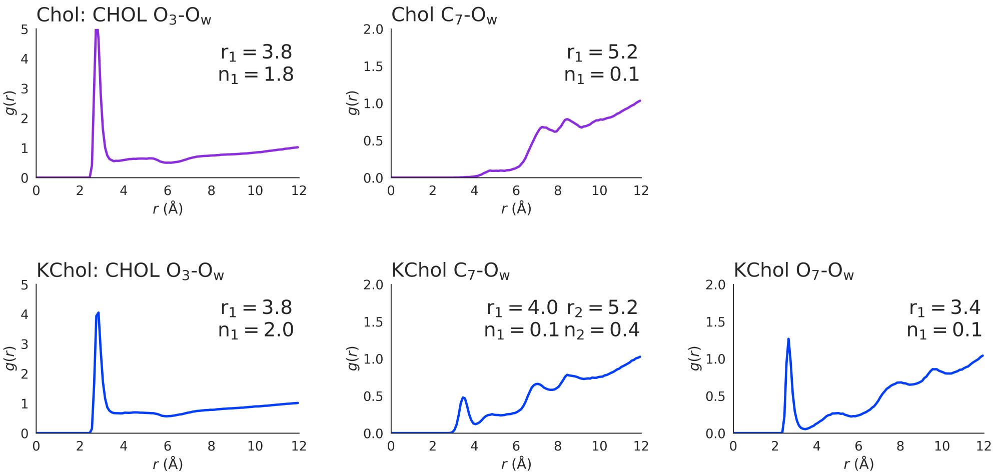

Figure S15. RDF of O_W atoms around the hydroxyl and ketone oxygen atoms of Chol and KChol.

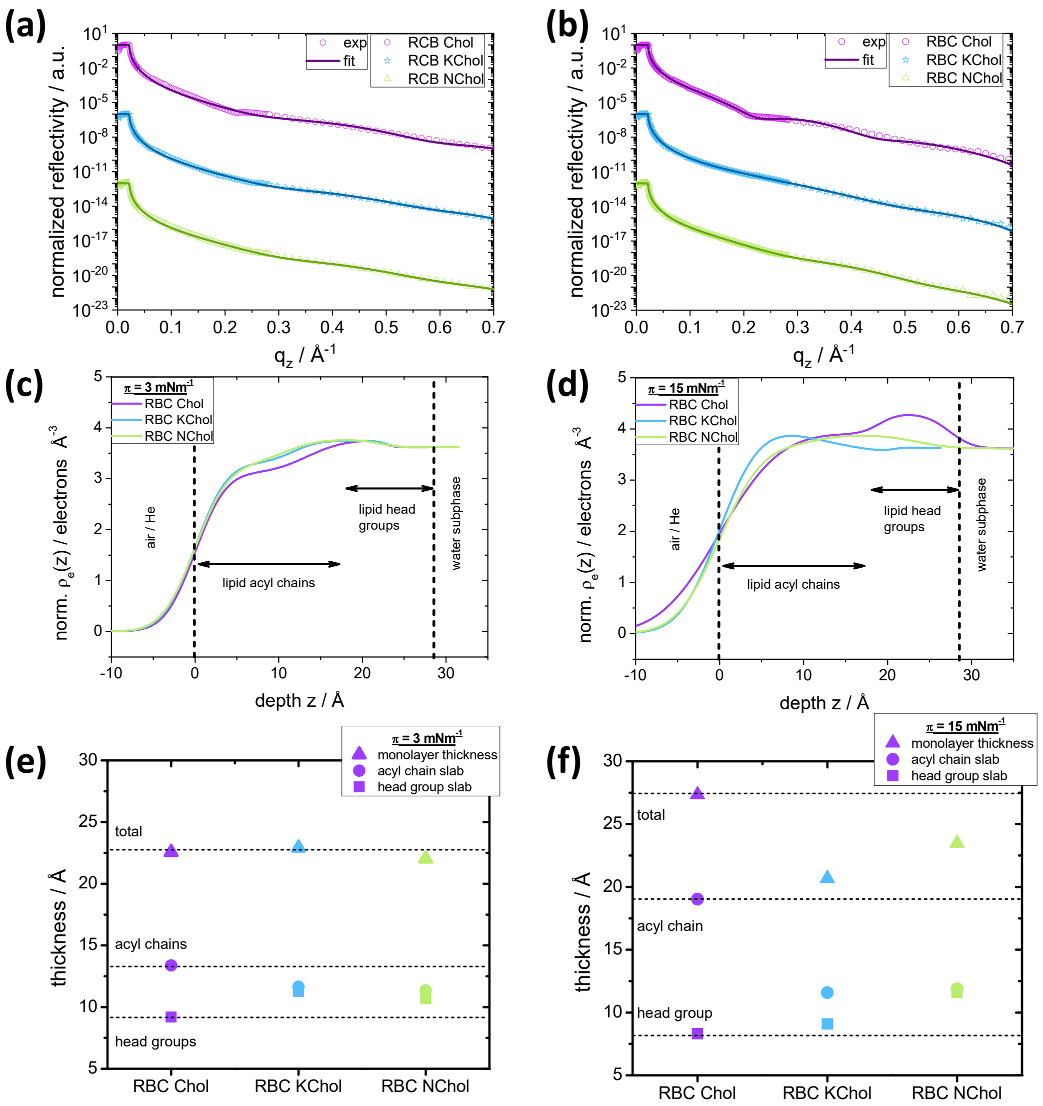

Figure S16. X-ray reflectivity at lower pressures. (a, b) Experimental (symbols) X-ray reflectivity curves of RBC Chol (violet), RBC KChol (blue) and RBC NChol (green) at 3 mN m^{-1} (a) and 15 mN m^{-1} (b) and best fits (lines) (the curves are shifted along the *y*-axis for clarity); (c, d) electron density profiles at the same pressures; (e, f) thicknesses of the monolayer and slabs (lipid headgroup, acyl chains, total) at the same pressures. The X-ray experiments were performed using aqueous TRIS buffer at pH = 7.4, 150 mM NaCl, 50 mM $CaCl_2$ subphase at 20 °C.

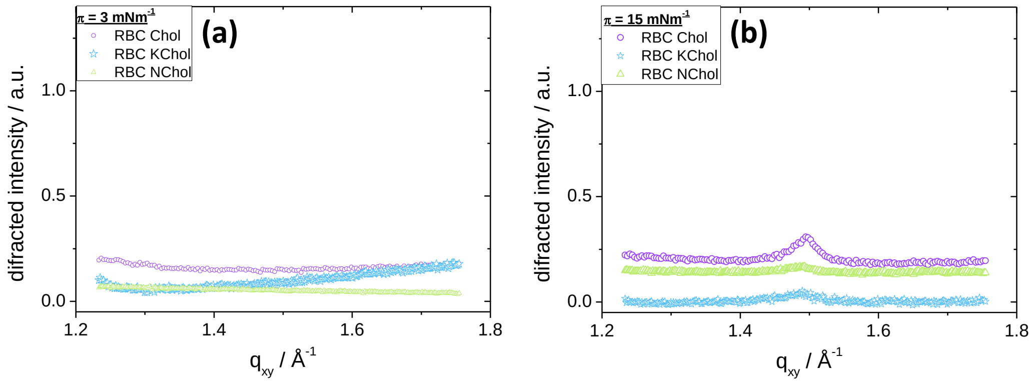

Figure S17. X-ray grazing incidence at lower pressures. GIXD curves for RBC Chol (violet), RBC KChol (blue) and RBC NChol (green) at 3 mN m^{-1} (a) and 15 mN m^{-1} (b). The X-ray experiments were performed using aqueous TRIS buffer at pH = 7.4, 150 mM NaCl, 50 mM $CaCl_2$ subphase at 20 °C.
